# Framework for personalized prediction of treatment response in relapsing-remitting multiple sclerosis: a replication study in independent data

**DOI:** 10.1186/s12874-024-02264-9

**Published:** 2024-06-24

**Authors:** Anna Maria Sakr, Ulrich Mansmann, Joachim Havla, Begum Irmak Ön, Begum Irmak Ön

**Affiliations:** 1 Institute for Medical Information Processing, Biometry and Epidemiology (IBE), Faculty of Medicine, LMU Munich, Marchioninistrasse 15, Munich, 81377 Germany; 2 Pettenkofer School of Public Health, Elisabeth-Winterhalter-Weg 6, Munich, 81377 Germany; 3 Institute of Clinical Neuroimmunology, University Hospital, LMU Munich, Marchioninistrasse 15, Munich, 81377 Germany

**Keywords:** Clinical prediction rule, Multiple sclerosis, Relapsing-Remitting, Registry data, Prognosis, Reproducibility of results

## Abstract

**Background:**

Individualizing and optimizing treatment of relapsing-remitting multiple sclerosis patients is a challenging problem, which would benefit from a clinically valid decision support. Stühler et al. presented black box models for this aim which were developed and internally evaluated in a German registry but lacked external validation.

**Methods:**

In patients from the French OFSEP registry, we independently built and validated models predicting being free of relapse and free of confirmed disability progression (CDP), following the methodological roadmap and predictors reported by Stühler. Hierarchical Bayesian models were fit to predict the outcomes under 6 disease-modifying treatments given the individual disease course up to the moment of treatment change. Data was temporally split on 2017, and models were developed in patients treated earlier (*n* = 5517). Calibration curves, discrimination, mean squared error (MSE) and relative percentage of root MSE (RMSE%) were assessed by external validation of models in more-recent patients (*n* = 3768). Non-Bayesian fixed-effects GLMs were also applied and their outcomes were compared to these of the Bayesian ones. For both, we modelled the number of on-therapy relapses with a negative binomial distribution, and CDP occurrence with a binomial distribution.

**Results:**

The performance of our temporally-validated relapse model (MSE: 0.326, C-Index: 0.639) is potentially superior to that of Stühler’s (MSE: 0.784, C-index: 0.608). Calibration plots revealed miscalibration. Our CDP model (MSE: 0.072, C-Index: 0.777) was also better than its counterpart (MSE: 0.131, C-index: 0.554). Results from non-Bayesian fixed-effects GLM models were similar to the Bayesian ones.

**Conclusions:**

The relapse and CDP models rebuilt and externally validated in independent data could compare and strengthen the credibility of the Stühler models. Their model-building strategy was replicable.

**Supplementary Information:**

The online version contains supplementary material available at 10.1186/s12874-024-02264-9.

## Background

In recent years, a wide spectrum of disease-modifying treatments (DMTs) became available for relapsing-remitting multiple sclerosis (RRMS) patients [1, 2]. The Multiple Sclerosis (MS) Therapy Consensus Group recommends choosing a DMT based on individual disease and personal characteristics, and the risk versus benefit profile of the therapy [2]. This endeavour is difficult due to the heterogeneous nature and course of RRMS, the multitude of DMTs investigated at the group level in selected trial populations that do not mainly represent real-world MS populations, and the rarity of head-to-head comparisons [3].

Multivariable prognostic models have been developed to predict the individual RRMS course, but most of them lack methodological quality or external validation, hampering their implementation in clinical practice [4–6]. Stühler et al. [7] proposed methodologically sound prediction models for personalizing DMT choice in RRMS patients based on their characteristics. They used real-world data from the German NeuroTransData registry (NTD) (https://www.neurotransdata.com/) which extend the clinical trial data by including heterogeneous MS patient profiles from a large number of different clinical sites. The selected target population consisted of adult RRMS patients under treatment with specific DMTs and whose disease is not progressed. We believe that this selected population could answer questions of medical relevance such as what could be a starting therapy or a subsequent therapy for a treated RRMS patient in need of a therapy change [3]. Based on the proposed models, Stühler et al. [7] provide guidance when choosing or switching to another DMT at least 6 months after diagnosis between 6 DMTs: dimethyl fumarate, fingolimod, glatiramer acetate, interferon beta1, natalizumab, and teriflunomide. The models included clinical and demographic variables commonly collected during routine care and predicted outcomes after the treatment switch given the patient history up to that switch. The outcomes were the number of on-therapy relapses and the occurrence of confirmed disability progression (CDP), which are widely used in clinical practice to monitor the disease and assess treatment effectiveness. Hierarchical Bayesian generalized linear models (GLMs) were fitted to data from adult RRMS patients registered in NTD until July 1, 2018. Model calibration and discrimination were evaluated under several internal validation schemes.

A prognostic model needs robustness and transportability to other patient settings before it is implemented into clinical practice. In this paper we looked on prognostic models which also include treatment as a factor and its interaction with other prognostic factors. Such a model is often called a treatment response prediction model. It should be developed on a representative data set of sufficient size. Estimating treatment interaction parameters requires higher patient numbers than estimating average treatment effects [8]. Such models reflect the prognosis observed in specific patient groups under specific treatment. They provide associations in such groups between treatment given and outcome observed. Formally, they are not causal models that would allow statements about changes in outcome if changes in treatment occur. This delicate distinction is often overlooked. In this paper we therefore used the term prognostic model and quantify associations between treatment history and patient features with respect to a future outcome. We avoid statements on optimal treatment effects for individual patients [9], because our approach may be impaired by unobserved confounding. For example, if a new drug is introduced on the market, changes from older drugs to the new drug may be motivated by market strategies of the respective company [10]. Effects observed after such switches are “prognostic” in terms that they reflect the effect of the drug and not interactions between drug and patient’s features.

Validation should assess discrimination: the model’s potential to differentiate between patients with and without favourable outcomes. Also, calibration needs to be demonstrated: agreement between predicted outcome probability under the received treatment and the outcomes observed frequencies. Good calibration avoids harmful effects as over- or under-treatment [11]. External validation is the preferred method to investigate discrimination and calibration. It ensures validity in a population other than that of the model development, confirming its generalisability [12].

The models in Stühler et al. [7] (referred to in our study as NTD models) were published as black boxes not accompanied by model coefficients, freely available tools, or instructions except the predictive factors they use. This hinders their implementation or independent validation. To our knowledge, they have not yet been externally validated. As the next best option, similar independent findings in a replication study following the same methodological approach and using the same medical input can increase confidence in these models. Hence, we aimed to (1) independently replicate the NTD model-building process to predict probabilities of being relapse-free and CDP-free in response to 6 DMTs in RRMS patients from a different setting, and (2) externally evaluate these replicated models by temporal validation.

## Methods

### Data source and participants

Since 2011, the real-world MS registry Observatoire Francais de la sclérose en plaque (OFSEP) documents patient records from 38 centres in France. It collects standardized data retrospectively during the first visit and prospectively thereafter [13–15]. A subset of the OFSEP dataset until December 15, 2021 (Supplementary Material (SM) Table S1) was transferred to us after approval by the OFSEP Steering Committee (0266). It contained 78 419 therapy cycles from 29 021 patients (SM Table S2). The Ethics Committee of LMU Munich (21-1174) also approved the project. OFSEP announced our study on their website (https://www.ofsep.org/fr/etudes/extval-phrend) and their patients could give project specific dynamic consent [16].

**Fig. 1:**
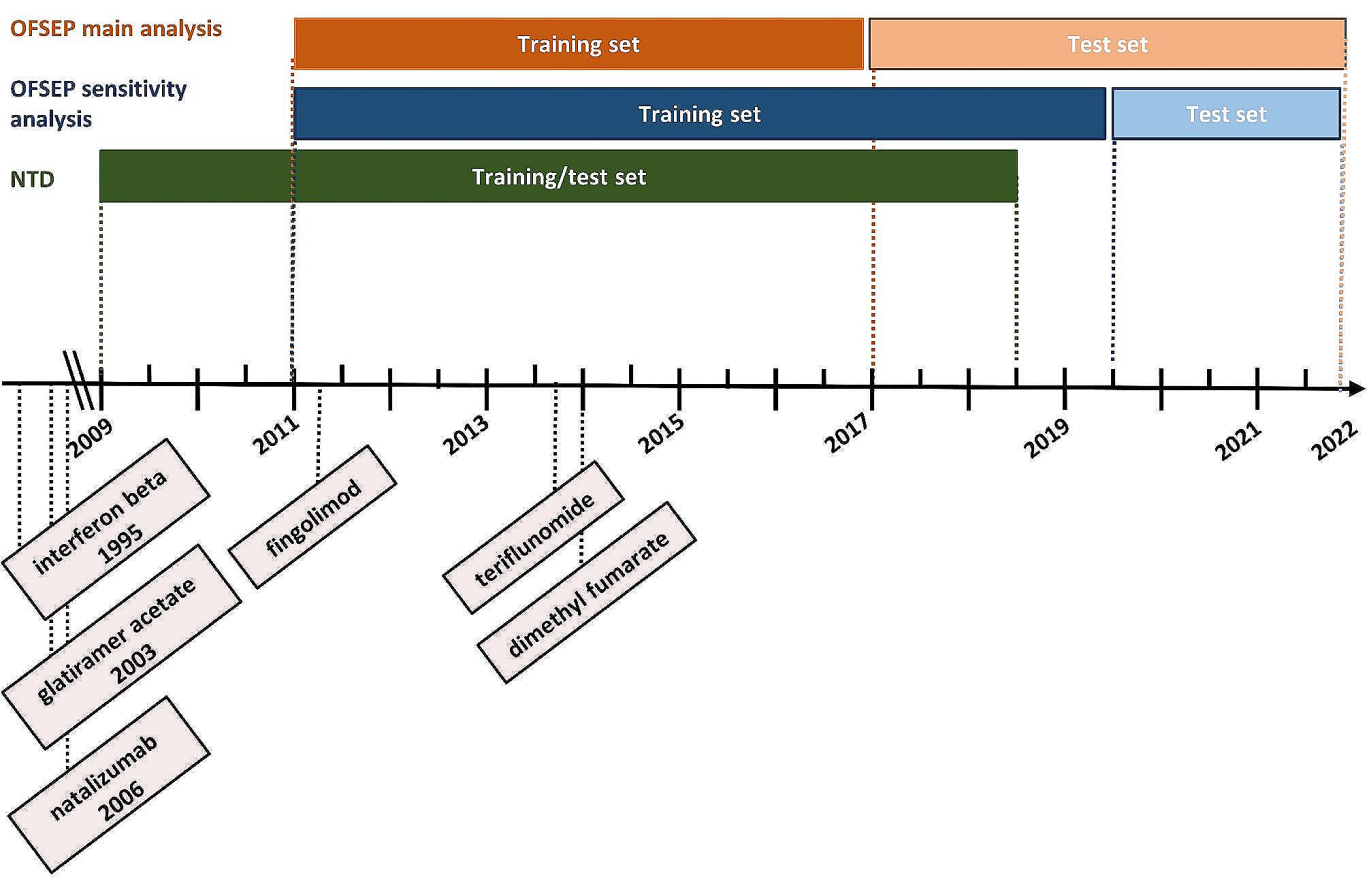
Time ranges of OFSEP (in main and sensitivity analysis sets) versus NTD study data and the European Medicines Agency approval timeline of 6 included disease modifying therapies

The transferred OFSEP dataset included heterogeneous patient profiles. Thus, we had to process the data to select a population of interest with quality documentation, which includes adult RRMS patients with a non-progressed disease and in need of therapy change. We derived the analysis set by replicating the data pre-processing by Stühler et al. [7]. This included quality criteria to ensure complete, valid, and consistent observations, and designing of therapy timelines (SM Table S2). We applied the same eligibility criteria to identify the targeted population and removed therapy cycles starting before the foundation of OFSEP (2011) rather than that of NTD (2008) (SM Table S3). Figure 1 shows the OFSEP versus NTD study data time ranges alongside the approval dates of the included DMTs, for informative purposes only.

### Variables

**Fig. 2:**
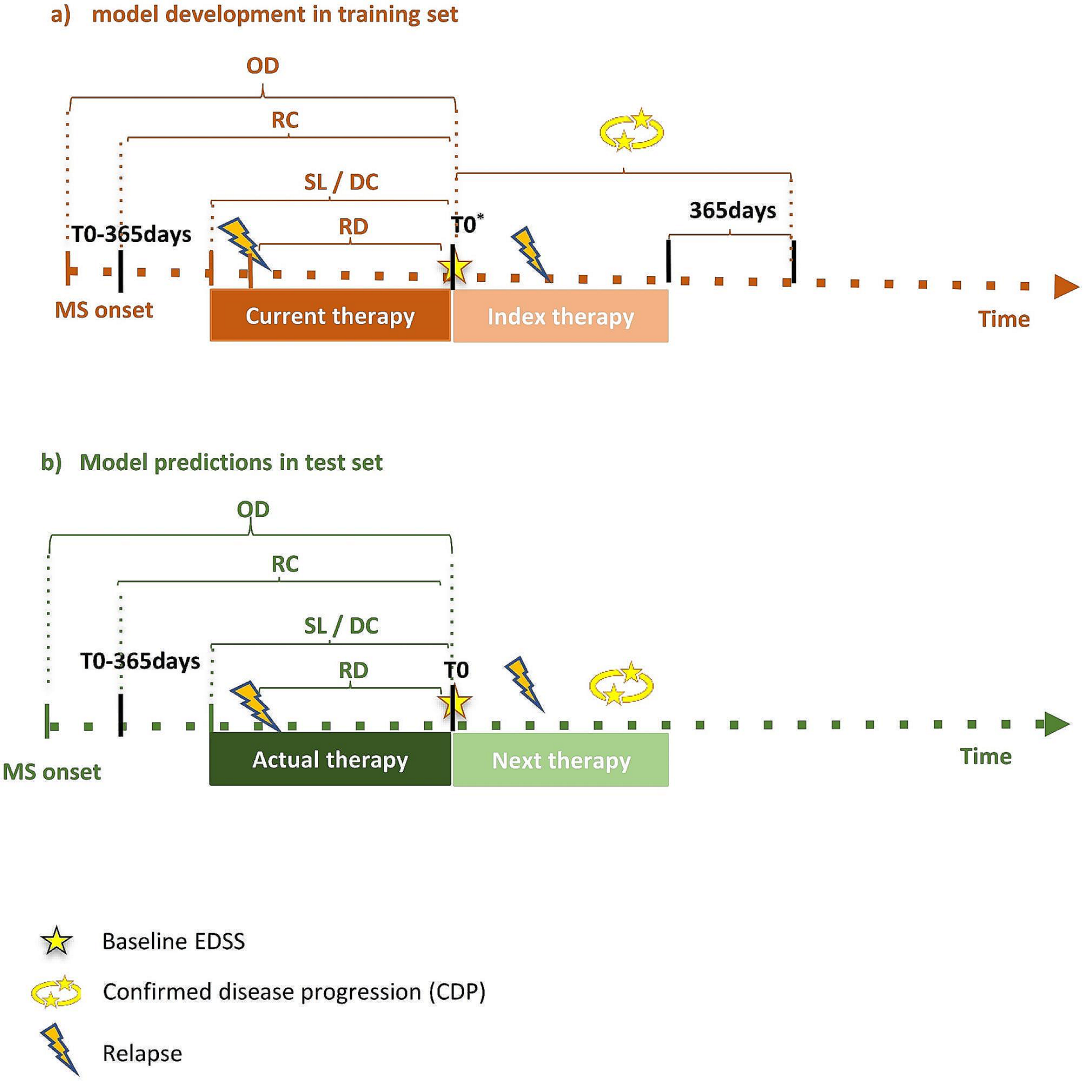
Example patient timelines demonstrating the study design. RD relapse distance, RC relapse count, OD onset distance, baseline EDSS Expanded Disability Status Scale. DC previous DMTs count, SL previous second-line DMT. Current and index therapies indicate a treatment switch. **a**: Timeline of a patient from the training set and variable assessment windows relative to the index therapy cycle, the start date of which is the baseline (T0). The outcomes were assessed during the index therapy. **b**: Timeline of a new patient from the test set with the predicted outcomes under the next possible therapy given their actual characteristics. The baseline denotes the time of treatment switch

We used 11 predictors and assessed all covariates before or at baseline (the start of the index therapy). A therapy cycle represents the period a patient spends on a specific DMT and is defined by a combination of start date, end date, and a DMT (Fig. 2). We defined and coded all variables in accordance with the NTD models (SM Figure S1 and Table S4). Current and index therapy cycles represent two DMTs forming a treatment switch, with their respective durations, where a decision to switch to another DMT is made for a patient on current therapy. The index therapy cycle follows the current therapy cycle and is the treatment actually switched to by the study sample. Effectiveness outcomes are measured during the index therapy cycle. Other included predictors were age, gender, the baseline Expanded Disability Status Scale (EDSS), the time since the last relapse, the number of relapses in the previous year, the time since MS onset, and finally the clinical site. The DMTs received previous to the index therapy cycle were taken into account not only as current therapy and its duration, but also the number of all prior DMTs taken, and whether these were second-line. The outcomes of interest were the number of on-therapy relapses and CDP occurrence during index therapy. CDP occurrence was defined according to the NTD working group based on the elevation of several EDSS measurements. It is at least a 0.5 (if baseline EDSS > 5.5) or 1 point increase in EDSS during the therapy, which was sustained by no decrease for the next 3 months and confirmed by the next EDSS measurement between 3 months after the EDSS increase and up to 12 months after the end of the therapy. Therapy cycles with no baseline or on-therapy EDSS measurements were classified as CDP-free. The confirmatory EDSS measurement had to be at least 3 months after a relapse (SM Figure S2).

### Sample size

The sample size calculation assumed similar discriminative performance as the NTD models. It used the outcome rates and test set C-indices as reported by Stühler et al. [7] in their Table S1.2 and their Table 7. The two-sided significance level was set to 5% and the power to 90% to test the alternative (C-index of 0.65) against the null-hypothesis (C-index of 0.6). Assuming that 25% of the study population experienced a relapse, the respective sample size was 1663 [17]. Also, assuming that 15% of the study population experienced a CDP, the sample size was 2618 to test the alternative (C-index of 0.6) against the null-hypothesis (C-index of 0.55) (SM Table S5).

### Statistical analysis methods

#### Missing data

We described missing data in the transferred and analysis datasets. Incomplete data was expected given the observational nature of the data source. We performed a complete case analysis similar to Stühler et al. [7].

#### Model development and validation

We fitted hierarchical Bayesian GLMs to the OFSEP data using the R package *Rstanarm* [18, 19]. The negative binomial distribution models the number of on-therapy relapses, the binomial distribution models CDP occurrence. A random intercept quantifies potential variability among centres. The duration of the follow-up was accounted for by the logarithm of index therapy duration (offset). All categorical variables were coded as nominal and the reference level for each variable was specified in Table S4. The model priors were weakly informative (default settings). Model diagnostics (see SM Box S1) ensured sampler convergence and reliable parameter estimation. Comparison of the model coefficient’s precision used the median absolute deviations (MAD). The absolute magnitude of the posterior model coefficients determines the predictor importance.

We split the data on January 1, 2017 based on the therapy start date. This specific date was chosen to have enough patients (approximately 60%/40%) having taken the 6 DMTs and to allow for comparable follow-up times in both datasets, as this can impact the outcome incidence. Temporal validation respects non-random variation between model development (training) and validation (test) datasets and validates the model on future (more recent) patients [20]. Ongoing therapy cycles were censored on the date of a patient’s last clinical assessment (EDSS measurement) occurring before the data extraction date. Therefore, we censored the observations in the training set on the date of the patients’ last EDSS measurement before the split date. This prevents data leakage by temporal overlap between training and test sets. Random selection of one therapy cycle per patient before splitting the data ensured independence of the training and test sets. We also internally evaluated the model performance via 10-fold cross-validation within the training set using out-of-sample predictions. We standardized continuous predictors using only the mean and standard deviation calculated in the training set [21].

#### Model performance

We assessed calibration and discrimination of both models by summarizing the mean and outcome-free proportions of posterior predictions for each patient while assuming an average centre effect (i.e., null random effect). We plotted calibration curves and estimated their intercept and slope. As Stühler et al. [7], we divided the predictions into 20 equally-populated bins and plotted the average observed versus predicted outcomes for every bin overall and per DMT. We assessed the models’ ability to discriminate those with and without the outcome by C-indices. The mean squared error (MSE) and the relative percentage of root MSE (RMSE%) assessed the overall model fit.

There were few methodological differences between our and Stühler et al. [7] approach (SM Table S6). Data processing and analyses were performed in R, version 4.2.2. (SM Box S2). We used TRIPOD checklist for reporting (SM Table S7).

#### Sensitivity analyses

We checked the robustness of our findings to a different sample size split (approximately 80%/20%), follow-up time, and a more recent test set by temporally splitting the data on another date: July 1, 2019. We also repeated the model-building and validation processes with non-Bayesian fixed effects GLMs. As for the Bayesian analysis, we modelled CDP occurrence with a binomial distribution and the number of relapses with a negative binomial distribution.

## Results

### Patient population

**Fig. 3:**
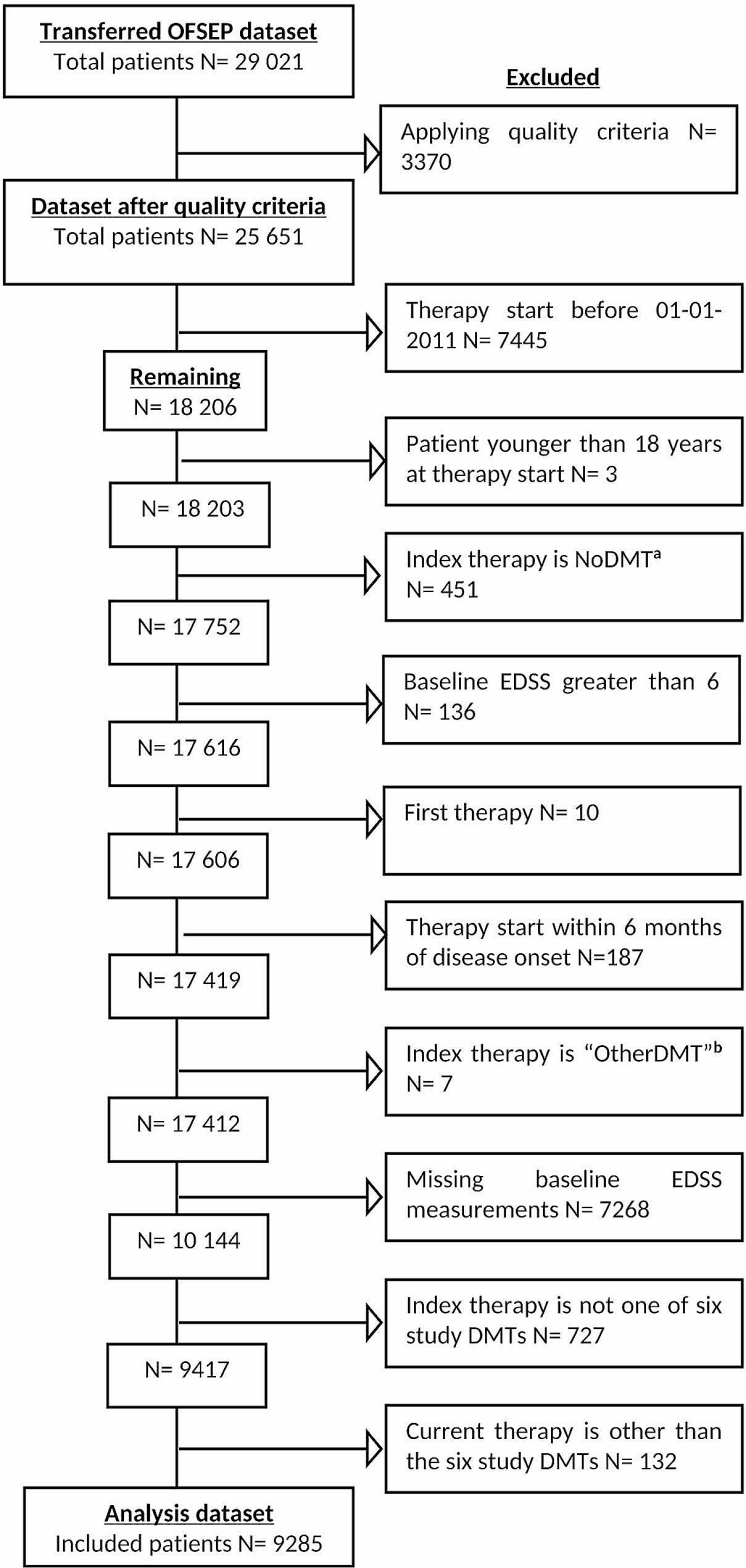
Participant flowchart from the transferred to the analysis dataset. DMT disease-modifying therapy; EDSS Expanded Disability Status Scale.^a^*Periods during which the patient received no therapy;*^*b*^*Periods during which therapy cycles of different DMTs were overlapping labeled as “OtherDMT”.*

In the transferred dataset, approximately 1% of the values were missing separately for the variables therapy start date, therapy end date, and EDSS measurement (alongside its associated date). In the analysis set, 38% of the therapy cycles were missing baseline EDSS (SM Table S8). After applying the inclusion criteria (Fig. 3), the analysis set (*n* = 9285) was split into training and test sets, with 5517 and 3768 patients, respectively.

The OFSEP training set (Table 1) consisted of 4155 (75%) female patients. At baseline, 1843 (33%) patients were aged between 31 and 40. As index therapy, 1381 (25%) patients received fingolimod and 556 (10%) patients received natalizumab. A total of 2411 (44%) patients had a baseline EDSS of 1.5 or less. A total of 2870 (52%) were not taking any DMT (current therapy is “NoDMT”) prior to their index therapy.

OFSEP training, test, and NTD training sets [7] contained at least 556, 284, and 281 therapy cycles for each DMT. The case-mix between the OFSEP training, OFSEP test, and NTD datasets compared well except for few characteristics. The median index therapy duration in the NTD population was longer: 1.92 compared to 1.37 years in the OFSEP training population, because of the censoring and temporal splitting of the OFSEP data. More patients in the OFSEP training population compared to the NTD population received teriflunomide as index therapy (17% versus 13%), had a baseline EDSS equal to 4 or higher (18% versus 15%), and had previously received second-line therapy (19% versus 12%).

The distribution of index therapy differed between OFSEP training and test datasets for teriflunomide (17% versus 25%) and interferon beta1 (16% versus 8%). The distribution of current therapy also differed for dimethyl fumarate (3% versus 9%), teriflunomide (1% versus 8%), glatiramer acetate (11% versus 6%) and interferon-beta1 (22% versus 12%). Median current therapy duration was higher in the training population compared to the test population (2.03 versus 1.63 years), as well as the proportion of patients with baseline EDSS equal to 4 or higher (18% versus 11%) and the onset distance respectively (7.5 versus 5.79 years), which may be an artefact caused by the temporal split.

The proportion of relapse-free patients (Table 2) was higher in the OFSEP test population (84%) compared to the OFSEP training population (77%), but the proportion of CDP-free patients was similar (92% and 91% respectively). The outcome rates were higher in the NTD dataset with the proportion of CDP-free patients at 84%, the median number of relapses at 0.

**Table 1:** Overview of predictors at baseline in the OFSEP training, test, and NTD training populations

Predictor	Category	Training (*N* = 5517) median (interquartile range; range) or count (%)	Test (*N* = 3768) median (interquartile range; range) or count (%)	NTD^a^ (*N* = 3119) median (interquartile range; range) or count (%)
Current therapy duration^b^		2.03 (4.26; 0.01–46.96)	1.63 (3.34; 0.04–51.82)	2.07 (4.2; 0.02–35.36)
Index therapy duration		1.37 (1.6; 0-5.91)	1.29 (1.83; 0-4.87)	1.92 (2.83; 0.08–9.39)
Onset distance^c^		7.5 (10.27; 0.5-54.96)	5.79 (9.6; 0.5-51.83)	6.38 (9.03; 0.10-47.18)
Relapse count		0 (1; 0–5)	0 (1; 0–4)	0 (1; 0–6)
Age	30 or younger	916 (17)	728 (19)	593 (19)
	31 to 40	1843 (33)	1382 (37)	967 (31)
	41 to 50	1724 (31)	974 (26)	1029 (33)
	51 or older	1034 (19)	684 (18)	530 (17)
Current therapy	dimethyl fumarate	150 (3)	333 (9)	94 (3)
	fingolimod	89 (2)	136 (4)	62 (2)
	glatiramer acetate	595 (11)	244 (6)	374 (12)
	interferon beta1	1227 (22)	443 (12)	811 (26)
	natalizumab	519 (9)	139 (4)	125 (4)
	teriflunomide	67 (1)	290 (8)	62 (2)
	NoDMT	2870 (52)	2183 (58)	1591 (51)
DMT count	0	1435 (26)	1177 (31)	717 (23)
	1	2057 (37)	1325 (35)	1528 (49)
	2	1149 (21)	729 (19)	561 (18)
	3 or more	876 (16)	537 (14)	312 (10)
Baseline EDSS	1.5 or less	2411 (44)	2040 (54)	1466 (47)
	2 to 2.5	1336 (24)	898 (24)	749 (24)
	3 to 3.5	759 (14)	434 (12)	437 (14)
	4 to 10	1011 (18)	396 (11)	468 (15)
Female		4155 (75)	2864 (76)	2339 (75)
Index therapy	dimethyl fumarate	1086 (20)	738 (20)	686 (22)
	fingolimod	1381 (25)	767 (20)	780 (25)
	glatiramer acetate	663 (12)	538 (14)	405 (13)
	interferon beta1	901 (16)	284 (8)	593 (19)
	natalizumab	556 (10)	488 (13)	281 (9)
	teriflunomide	930 (17)	953 (25)	405 (13)
Relapse distance	less than 0.25	407 (7)	234 (6)	530 (17)
	0.25 to 0.99	1961 (36)	1175 (31)	998 (32)
	1 to 2.99	1561 (28)	1173 (31)	530 (17)
	3 or more	1588 (29)	1186 (31)	749 (24)
Second-line therapy^d^		1051 (19)	518 (14)	374 (12)

OFSEP Observatoire Francais de la sclérose en plaque; NTD NeuroTransData; DMT disease-modifying therapy; EDSS Expanded Disability Status Scale; Continuous variables (in years) summarized by median (interquartile range; range); categorical variables by count (%);^a^Counts derived from summary measures, ^b^The high maximum duration of current therapy is due to therapy-free periods (“NoDMT”) before the index therapy (SM Figures S3 and S4).^c^Onset distance in this study is the time from disease onset till start of index therapy as compared to Diagnosis distance in Stühler et al., (7) which is based on time from MS diagnosis.^d^As in Stühler et al. (7) alemtuzumab, cyclophosphamide, fingolimod, mitoxantrone, natalizumab, ocrelizumab, and rituximab

**Table 2:** Overview of outcomes in the OFSEP training, test, and NTD training populations

Outcome	Training (*N* = 5517) median (interquartile range; range) or count (%)	Test (*N* = 3768) median (interquartile range; range) or count (%)	NTD^a^ (*N* = 3119) median (interquartile range; range) or count (%)
CDP-free	5005 (91)	3449 (92)	2620 (84)
Relapse-free	4225 (77)	3177 (84)	Not reported
Number of relapses	0 (0; 0–6)	0 (0; 0–4)	0 (1; 0–7)

OFSEP Observatoire Francais de la sclérose en plaque; NTD NeuroTransData; CDP confirmed disease progression; Number of relapses summarized by median (interquartile range; range), the rest by count (%).^a^Derived from Stühler et al. [7]

### Model diagnostics

Using the identical variable coding and modelling approach as NTD, we compared the precision of the regression coefficients by MADs. A high Spearman correlation between the MADs from our and the NTD models (CDP: 0.96, Relapse: 0.98) demonstrated high agreement in precision ranking. The predictor current duration and its interactions with current therapy categories, especially with teriflunomide, had large MADs in both models and both datasets (OFSEP and NTD), indicating general uncertainty in their estimation (SM Tables S9 to S11).

### Model performance

The data for building our models was very informative: 56 degrees of freedom, with 23 and 9.1 events per variable for the relapse and CDP outcomes respectively in the training set. We present in Table 3 an overall comparison of the predicted and observed mean number of relapses and proportion with CDP. In the cross-validation, the mean predicted number of relapses was higher than the observed 0.49 (range 0 to 42.2) and 0.33 (range 0 to 6) respectively. Whereas for CDP, the predicted proportion with CDP (mean) was close to the observed proportion with CDP 0.1 (range 0 to 0.72) and 0.09 (range 0 or 1) respectively. In the temporal validation, the mean predicted number of relapses was 0.32 (range 0 to 4.4) compared to the observed of 0.2 (range 0 to 4), and lower in relapse-free patients (0.3) than in those who relapsed (0.435). For 79% of the patients, the model predicted a relapse-free therapy cycle (observed 84%). CDP-free therapy cycles were predicted for 91% of the patients (observed 92%). The predicted individual CDP risk was similar to the observed one with 0.09 and 0.08 respectively. It ranged from 0.0 to 0.56 (mean of 0.084 in patients without and 0.159 in those with CDP occurrence).

For the relapse outcome (Table 3), the cross-validation revealed a higher error (MSE 1.294) but also a higher discriminatory power (C-index 0.7, 95%CI 0.684 to 0.715) in the OFSEP model compared to the NTD model (MSE 0.755 and C-index 0.646, 95%CI 0.645 to 0.647), and an RMSE of 158%. The MSE (0.326), RMSE (113%) and the C-index (0.639, 95%CI 0.616 to 0.661) were lower in temporal validation of the OFSEP model compared to its cross-validation. The performance in the random-split validation of the NTD model had also been worse than its performance in cross-validation (MSE 0.784, C-Index 0.608). The calibration curve in the temporally-split test set had an intercept of -0.386 (95%CI -0.479 to -0.293) and a slope of 0.501 (95%CI 0.41 to 0.593), hinting at miscalibration.

For the CDP outcome, the cross-validation performance of the OFSEP model was better than that of NTD both in terms of error (MSE 0.081 versus 0.125) and discrimination (C-index 0.737, 95%CI 0.718 to 0.757 versus 0.582, 95%CI 0.580 to 0.584). The OFSEP model also performed much better in temporal validation (MSE 0.072; C-index 0.777, 95%CI 0.754 to 0.798) compared to the NTD model in random-split validation (MSE 0.131; C-index 0.554). The RMSE was 99% in both validations. The calibration curve in the temporally-split test set was close to ideal with an intercept of -0.079 (95%CI -0.198 to 0.04) and a slope of 1.097 (95%CI 0.948 to 1.246).

**Table 3:** Performance measures of the OFSEP and NTD models

Outcome	Performance measure (95% CI)	OFSEP cross-validation^a^ *N* = 5517	OFSEP temporal validation *N* = 3768	NTD cross-validation^a^ *N* = 3119	NTD random-split validation *N* = 314
CDP	Calibration intercept	-0.096 (-0.192 to-0.001)	-0.079 (-0.198 to 0.04)	Not reported	Not reported
	Calibration slope	0.802 (0.705 to 0.899)	1.097 (0.948 to 1.246)	Not reported	Not reported
	C-index	0.737 (0.718 to 0.757)	0.777 (0.754 to 0.798)	0.582 (0.580 to 0.584)^c^	0.554
	MSE^b^	0.081	0.072	0.125	0.131
	RMSE%	99%	99%	96%^d^	Not reported
	Predicted proportion with CDP (range)	0.1 (0 to 0.72)	0.09 (0 to 0.56)	Not reported	Not reported
	Observed proportion with CDP (range)	0.09 (0 or 1)	0.08 (0 or 1)	0.16 (0 or 1)	Not reported
Relapse	Calibration intercept	-0.01 (-0.079 to 0.06)	-0.386 (-0.479 to -0.293)	Not reported	Not reported
	Calibration slope	0.615 (0.557 to 0.673)	0.501 (0.41 to 0.593)	Not reported	Not reported
	C-index	0.7 (0.684 to 0.715)	0.639 (0.616 to 0.661)	0.646 (0.645 to 0.647)^c^	0.608
	MSE	1.294	0.326	0.755	0.784
	RMSE%	158%	113%	99%^d^	Not reported
	Mean predicted number of relapses (range)	0.49 (0 to 42.2)	0.32 (0 to 4.4)	Not reported	Not reported
	Mean observed number of relapses (range)	0.33 (0 to 6)	0.20 (0 to 4)	0.43 (0 to 7)	Not reported

OFSEP Observatoire Francais de la sclérose en plaque; NTD NeuroTransData; CDP confirmed disease progression; CI confidence interval; MSE mean squared error; RMSE% relative percentage of the root mean squared error.^a^The reported measures are estimated from out-of-sample predictions.^b^Equivalent to the Brier score for binary outcomes.^c^95% CI and^d^RMSE% calculated respectively from the standard errors and outcome summaries reported by Stühler et al. [7]. They split the data randomly into training (90%) and test (10%) sets. We split the data on 2017 into training and test sets. 10-fold cross-validations in both studies were performed in training sets.

**Fig. 4:**
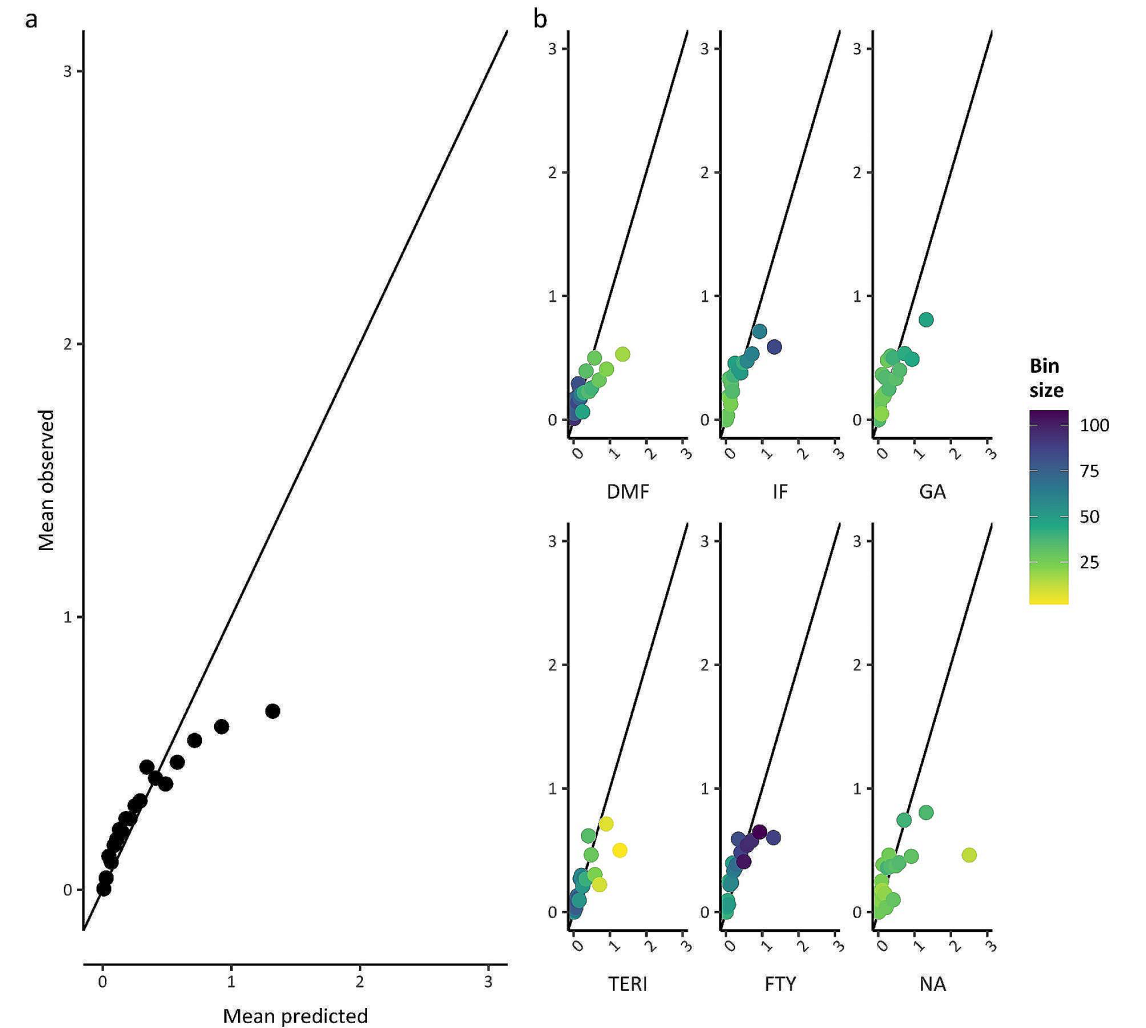
Calibration plots of the relapse model in the training set. Shown is the mean observed versus mean predicted number of relapses per prediction bin using out-of-sample-predictions: panel **a**: Overall (*N* = 275 or 276 patients per bin), panel **b**: for each DMT. DMF dimethyl fumarate; FTY fingolimod; GA glatiramer acetate; IF interferon beta1; NA natalizumab; TERI teriflunomide

**Fig. 5:**
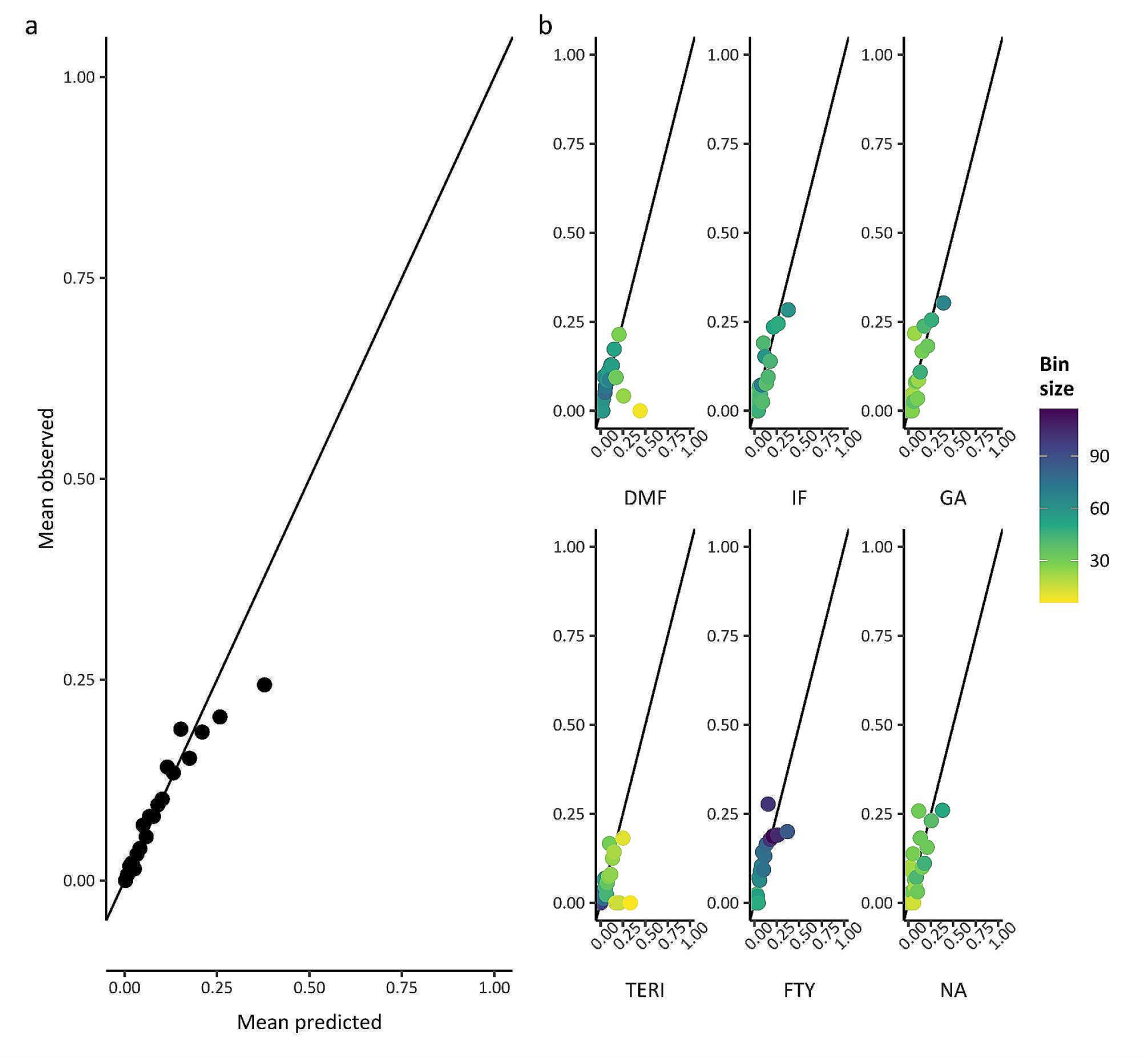
Calibration plots of the confirmed disease progression (CDP) model in the training set. Shown is the mean observed versus mean predicted proportion of CDP per prediction bin using out-of-sample-predictions: panel **a**: Overall (*N* = 275 or 276 patients per bin), panel **b**: for each DMT. DMF dimethyl fumarate; FTY fingolimod; GA glatiramer acetate; IF interferon beta1; NA natalizumab; TERI teriflunomide

Overall and DMT-specific calibration plots from cross-validation (Figs. 4 and 5) showed a similar trend to those reported for the NTD models (Fig. 3 in Stühler et al. [7]). We could not compare the calibration plots from test set validation (SM Box S3 and Figures S5-S8) that were not reported for the NTD models. Our relapse model over-predicted the number of relapses for patients with more observed relapses, both overall and per DMT. The CDP model had good overall calibration.

### Sensitivity analyses

When the data was split in 2019, the training and test sets contained 8159 and 1236 patients, respectively (SM Tables S12 to S14). Compared to the main analysis, the median index therapy duration was longer in the training (1.74 versus 1.37 years) whereas shorter in the test (0.77 versus 1.29 years) populations. Both outcomes were more frequent in the training population. The calibration of the CDP model in the 2019 test set was worse than that of the main analysis, showing moderate predictions compared to the observed outcome probability (calibration slope 1.778) but it had higher discrimination (C-index 0.833). The 2019 test set performance of the relapse model was slightly better than in the main analysis. The non-Bayesian fixed effect GLMs performed similarly to the hierarchical Bayesian models for both outcomes but, as expected, estimated in absolute values much larger coefficients mainly for the predictors with high MADs in the main analysis: current therapy, current duration, and their interaction terms (SM Figures S9, S10, and Table S15).

## Discussion

Focusing on the model’s accuracy (calibration and discrimination) and generalisability (reproducibility and transportability) is key to evaluating the performance of prediction models [22, 23]. In our study, we developed and validated models according to the processes and criteria described by Stühler et al. [7] on an independent and representative dataset from a French MS registry and assessed their accuracy and generalizability by a temporal split of the data at hand. Because Stühler et al. [7] presented black box models without details of their internal structure, we replicated the model-building with assistance from the NTD working group.

For the replication, we used predictors (content and coding) identical to the NTD models and reconstructed as loyally as possible all aspects of the data pre-processing and modelling. Finally, we implemented identical performance measures. While NTD assessed performance using a random data split, we validated our models by a deterministic temporal split. This mimics a realistic situation where a model is intended to be applied to future patients. A random split ignores the time structure like changes over time in the severity of the disease, aspects of care, diagnostic criteria to detect the disease, and availability and acceptance of newer treatments. A temporal split of an existing dataset is considered a form of external validation, whereas a random split is considered to be an internal validation scheme (20).

Our replication study showed that the developed OFSEP relapse model was miscalibrated in both the cross- and test set validation schemes. In terms of discrimination, the OFSEP relapse model performed better than the NTD model in both validation schemes (C-index OFSEP 0.7 and 0.639 versus NTD 0.646 and 0.608). The developed OFSEP CDP model showed good calibration and discrimination both in the patient set it was developed in and in more recently treated patients. Also, the discriminative performance of the OFSEP CDP model was better than its NTD counterpart in the respective validations (C-index OFSEP 0.737 and 0.777 versus NTD 0.582 and 0.554). The NTD working group regularly improves their models by incremental learning. They used quarterly updates of the NTD database with new patients [24] and reported changes in the C-indices overtime, which we interpreted as convergence to our C-indices. Therefore, our report is not able to compare the OFSEP results to the current (based on the learning from new data) but to the starting NTD models.

Furthermore, we could not validate the exact NTD models because the predictor weights were not published, and we cannot assume similarity to estimated model coefficients from our study. We only know that we used the same predictors and modelling approach. Based on reported MADs, we could compare the precision of the model coefficients and implicitly the information content of the data with the respective prognostic features. We observed similar structures reflected by high correlation, implying similar ranking. Nevertheless, we caution against the interpretation of the predictor importance assessment presented in Table S9, because it is based solely on the absolute values of the variables’ coefficients. A sensitive method such as permutation importance would take into account how each predictor impacts the models’ performance (i.e. MSE).

Our main contribution to the validation of the NTD strategy is the demonstration that their modelling approach worked in temporal split validation, which increases the case-mix heterogeneity between the training and test populations [25]. Thus, our results support the relevance of the NTD modelling approach for new patients encountered in clinical practice.

Our sensitivity analysis showed that the predictive ability of the non-Bayesian fixed-effects GLMs were nearly identical to the complex hierarchical Bayesian models. This confirms an often made finding that compared to complex models, standard approaches may give predictions of comparable quality by allowing a much simpler model communication and implementation [26].

This is the first external validation study for the replicated NTD models. To our knowledge, this is also the first time that any prediction model for MS patients was validated by an independent author team other than the creators of the respective model. A recent systematic review of prognostic prediction models in patients with MS [4] (accepted for publication) found that external validation studies are rare, and research activities should concentrate more on rigorously validating the existing prognostic instruments rather than developing new ones.

We had to make technical assumptions to replicate the coarsely described model-building and validation process. Despite this drawback, the findings from both studies align to a great extent. We did not explore the net benefit of our models. Nor did we investigate the robustness of the CDP definition, where patients with missing EDSS measurements were interpreted as CDP-free.

The large and representative sample, and the demonstration of geographical and temporal transportability of the CDP model and of the discriminative ability of the relapse model are the strengths of our study. The interpretability of the statistical methods and the easy applicability of the models in clinical practice were the strengths of the NTD models which also apply to the models in our study. This replication study is also a recalibration because we used the same predictors as in the NTD models but re-estimated respective coefficients using a different dataset. The miscalibration of the relapse model at internal validation hints at potential model misspecification, e.g., missing important predictors.

The analyses performed by NTD and by us describe the association between treatment and outcome in specific patient groups. It is conceivable that our models may naively be used to predict therapy effects in patient groups that have never received the therapy in question. We could identify such subgroups that were not considered for certain treatments in the OFSEP data. It is unclear if the same is the case in the NTD data. We would also like to caution against interpreting the association analyses causally: the proposed treatment does not necessarily cause the optimal possible effect. With the concise set of factors used in the models, there may even exist unobserved time-dependent confounding [27].

## Conclusion

We built association-based prediction models following the NTD modelling strategy and assessed their quality in the French OFSEP population. We found comparable and sometimes superior performance in our results. Therefore, the NTD modelling strategy was in principle replicable. Yet, we could not assess whether the application of the exact NTD models to the OFSEP data (full transportability) would perform similarly. Further research on the NTD modelling strategy could involve recalibration to new patients, simplification of the modelling method, updating with other common predictors, such as from magnetic resonance images, and assessment of the effect of differences in predictor measurement between our study and the clinical setting before its use, which could impact the model’s performance at application [28]. We implemented the NTD model-building idea in a new healthcare setting which proved to be successful but pointed to potential limitations.

### Electronic supplementary material

Below is the link to the electronic supplementary material.

Supplementary Material 1

## Data Availability

We endorse FAIR data and code sharing for reproducible research. However, the data that support the findings of this study is not publicly available due to legal reasons and can be obtained on request from OFSEP. The R code used to process and analyse the data is openly available in Gitlab at https://gitlab.lrz.de/asakr/repl_phrend.

## Abbreviations

CDP: Confirmed disability progression
CI: Confidence interval
DMF: Dimethylfumarat
DMT: Disease-modifying therapy
EDSS: Expanded Disability Status Scale
FTY: Fingolimod
GA: Glatirameracetat
GLM: Generalized linear model
IF: Interferon-ß1
LMU: Ludwig-Maximilians University
MAD: Median absolute deviation
MS: Multiple sclerosis
MSE: Mean squared error
NA: Natalizumab
NTD: NeuroTransData
OFSEP: Observatoire Francais de la sclérose en plaque
RRMS: Relapsing-remitting multiple sclerosis
SM: Supplementary material
TERI: Teriflunomide
