# Supplementary material for: Framework for personalized prediction of treatment response in relapsing-remitting multiple sclerosis: a replication study in independent data
Source: BMC Med Res Methodol. 2024 Jun 24;24:138. doi: 10.1186/s12874-024-02264-9 (PMC11194862; doi:10.1186/s12874-024-02264-9)
Supplement: Supplementary file 1 — Supplementary Material 1 [file 12874_2024_2264_MOESM1_ESM.docx]

**Supplementary Material to:**

**Framework for Personalized Prediction of Treatment Response in Relapsing-Remitting Multiple Sclerosis: A Replication Study in Independent Data**

Authors: Anna Maria Sakr^1,2^, Ulrich Mansmann^1,2^, Joachim Havla^3^, Begum Irmak Ön^1,2^

Affiliations:

1. Institute of Medical Information Processing, Biometry and Epidemiology (IBE), Faculty of Medicine, LMU Munich, Marchioninistrasse 15, 81377 Munich, Germany
2. Pettenkofer School of Public Health, Elisabeth-Winterhalter-Weg 6, 81377 Munich, Germany
3. Institute of Clinical Neuroimmunology, University Hospital, LMU Munich, Marchioninistrasse 15, 81377 Munich, Germany

Corresponding author: Anna Maria Sakr; anna.sakr@ibe.med.uni-muenchen.de

[**Table S1** Filtration criteria applied by OFSEP for this study. 3](#_Toc144214074)

[**Table S2** Quality criteria applied to the transferred OFSEP dataset. 4](#_Toc144214075)

[**Table S3** Inclusion criteria applied to derived analysis dataset. 5](#_Toc144214076)

[**Fig.S1** Transformation of the Observatoire Francais de la sclérose en plaque (OFSEP) dataset to derive the predictors. 6](#_Toc144214077)

[**Table S4** Predictor definitions 7](#_Toc144214078)

[**Fig.S2** Confirmed disability progression (CDP) definition. 8](#_Toc144214079)

[**Table S5** Required sample size in different scenarios assuming b=1 and for 90% power, alpha=0.05 in a two-sided test. 9](#_Toc144214080)

[**Box S1** Model development and diagnostics 10](#_Toc144214081)

[**Table S6** Methodological differences between this study and that of Stühler et al 11](#_Toc144214082)

[**Table S7** TRIPOD checklist 13](#_Toc144214083)

[**Table S8** Missing data in the transferred and the analysis datasets 15](#_Toc144214084)

[**Fig.S3** Duration of current therapy in the training set (N=5517). 16](#_Toc144214085)

[**Fig.S4** Duration of index therapy in the training set (N=5517). 17](#_Toc144214086)

[**Table S9** Most important predictors 18](#_Toc144214087)

[**Table S10** Posterior coefficients, 95% credible intervals and diagnostic measures of the relapse model. 19](#_Toc144214088)

[**Table S11** Posterior coefficients, 95% credible intervals and diagnostic measures of the CDP model 22](#_Toc144214089)

[**Fig.S5** Calibration plot of the relapse model in the test set. 25](#_Toc144214090)

[**Fig.S6** Calibration plot of the confirmed disease progression (CDP) model in the test set**.** 26](#_Toc144214091)

[**Fig.S7** Loess calibration plot of the relapse model in the test set. Observations are divided into 20 groups by their observed relapse. 27](#_Toc144214092)

[**Fig.S8** Loess calibration plot of the confirmed disease progression (CDP) model in the test set. Observations are divided into 20 groups by their observed CDP. 28](#_Toc144214093)

[**Table S12** Overview of predictors at baseline in the sensitivity analysis of splitting the dataset in 2019. 29](#_Toc144214094)

[**Table S13** Overview of outcomes in the sensitivity analysis of splitting the dataset in 2019 30](#_Toc144214095)

[**Table S14** Performance measures in the sensitivity analysis of splitting the dataset in 2019. 31](#_Toc144214096)

[**Fig.S9** Confirmed disease progression (CDP) model standardized parameters estimated in the main versus the sensitivity analysis. 32](#_Toc144214097)

[**Fig.S10** Relapse model standardized parameters estimated in the main versus the sensitivity analysis. 33](#_Toc144214098)

[**Table S15** Performance measures in the sensitivity analysis with the fixed effects generalized linear models 34](#_Toc144214099)

**Table S1** Filtration criteria applied by OFSEP for this study.

| **Description** | **Patient count** | **Patient count loss** |
| --- | --- | --- |
| OFSEP Export 2021-12-15 | 82 814 | 0 |
| Removal of duplicates | 75 169 | -7645 |
| Removal of NMOSD, AQP4+, MOG+ | 73 166 | -2003 |
| Removal of patients without MS onset date | 71 546 | -1620 |
| Removal of patients with inconsistencies in neurological episodes | 71 534 | -12 |
| Removal of patients with unconfirmed extreme age (=0 or >75) at MS onset | 71 478 | -56 |
| Removal of patients with age < 18 at MS onset | 68 361 | -3117 |
| Selection of patients with treatment studied^a^ | 36 665 | -31 696 |
| Selection of patients with RRMS at first treatment initiation | 31 865 | -4800 |
| Selection of patients starting treatment after 6 months after MS onset | 29 236 | -2629 |
| Selection of patients with no EDSS>6 at treatment initiation (at the EDSS the closer to initiation among them EDSS at +/- 3 months of initiation) | 29 021 | -215 |
| Removal of centres with only one patient | 29 021 | 0 |
| Total | 29 021 | -53 793 |
| *OFSEP Observatoire Francais de la Sclerose en Plaque, NMOSD Neuromyelitis Optica Spectrum Disorders, AQP4+ Aquaporin-4 seropositive patients, MOG+ Myelin Oligodendrocyte Glycoprotein seropositive patients, MS multiple sclerosis, RRMS relapsing-remitting multiple sclerosis, EDSS Expanded Disability Status Scale.^a^Studied treatments are dimethyl fumarate, fingolimod, glatiramer acetate, interferon beta, natalizumab, and teriflunomide.* | | |

# **Table S2** Quality criteria applied to the transferred OFSEP dataset.

| **Description** | **Patients**  **count** | **Patient**  **count loss** | **Therapy cycles**  **(TC)** | **TC**  **loss** |
| --- | --- | --- | --- | --- |
| Original entries | 29 021 |  | 78 419 |  |
| Remove duplicate observations | 29 021 | 0 | 78 350 | -69 |
| Remove non-RRMS patients at treatment initiation | 27 811 | -1210 | 74 921 | -3429 |
| Remove patients with therapy cycles used in clinical studies | 27 048 | -763 | 71 862 | -3059 |
| Remove patients with missing MS onset date | 27 048 | 0 | 71 862 | 0 |
| Remove patients with missing birth date | 27 048 | 0 | 71 862 | 0 |
| Remove patients/therapy cycles with birth, treatment start or end date, EDSS, relapse or MS onset date after data extraction date | 27 047 | -1 | 71 859 | -3 |
| Remove patients with birth date before 1916 | 27 047 | 0 | 71 859 | 0 |
| Remove therapy cycles with start, end, EDSS, relapse or MS onset date before birth date | 27 047 | 0 | 71 859 | 0 |
| Remove patients that have a therapy cycle with missing start date | 26 492 | -555 | 69 799 | -2060 |
| Remove patients with a therapy cycle end date before its start date | 26 492 | 0 | 69 799 | 0 |
| Remove therapy cycles corresponding to patients with no EDSS visits | 25 898 | -594 | 68 776 | -1023 |
| Remove therapy cycles with start date after last visit (EDSS date) | 25 659 | -239 | 67 703 | -1073 |
| Remove therapy cycles that were stopped within a day | 25 653 | -6 | 67 385 | -318 |
| Merge same therapy cycles with the same start and/or end dates | 25 653 | 0 | 67 360 | -25 |
| Add missing end dates | 25 653 | 0 | 67 360 | 0 |
| Remove total overlap with NoDMT | 25 653 | 0 | 67 360 | 0 |
| Merge overlapping same therapy cycles | 25 653 | 0 | 64 935 | -2425 |
| Merge overlapping different therapy cycles and label as “OtherDMT” | 25 653 | 0 | 64 011 | -924 |
| Add “NoDMT” cycles into gaps where no therapy cycle was recorded, including between MS onset and first therapy cycle | 25 653 | 0 | 119 375 | 55 364 |
| Remove “NoDMT” cycles after DMT with duration less than 92 days (wash out phase) | 25 653 | 0 | 103 295 | -16 080 |
| Merge subsequent different therapy cycles and fill therapy gaps of duration less than 92 days | 25 653 | 0 | 100 918 | -2377 |
| Remove discrepant EDSS measurements on the same date | 25 653 | 0 | 100 918 | 0 |
| Remove therapy cycles corresponding to patients with no EDSS visits, after censoring therapy cycles to their last EDSS visit =< 2017-01-01. | 25 651 | -2 | 95 531 | -5387 |
| *OFSEP Observatoire Francais de la sclérose en plaque; EDSS Expanded Disability Status Scale; DMT disease-modifying therapy; MS multiple sclerosis. After censoring the data on 2017, we derived the analysis dataset.* | | | | |

# **Table S3** Inclusion criteria applied to derived analysis dataset.

| **Description** | **Patients**  **count** | **Patient**  **Count loss** | **Therapy cycles**  **(TC)** | **TC**  **loss** |
| --- | --- | --- | --- | --- |
| Filter: Therapy cycle stopped within a day | 25 651 | 0 | 95 161 | -370 |
| Filter: Therapy cycle started before 2011-01-01 | 18 206 | -7445 | 45 534 | -49 627 |
| Filter: Therapy cycle started when patient was under 18 | 18 203 | -3 | 45 471 | -63 |
| Filter: Index therapy is “NoDMT” | 17 752 | -451 | 32 330 | -13 141 |
| Filter: EDSS at start of therapy cycle > 6 | 17 616 | -136 | 31 851 | -479 |
| Filter: Observed ARR is > 12 | 17 616 | 0 | 31 851 | 0 |
| Filter: First therapy cycle^a^ | 17 606 | -10 | 31 786 | -65 |
| Filter: Therapy cycle that started within 6 months after disease onset | 17 419 | -187 | 30 211 | -1575 |
| Filter: Index therapy is “OtherDMT” | 17 412 | -7 | 29 983 | -228 |
| Filter: Missing EDSS measurement at index therapy onset | 10 144 | -7268 | 13 839 | -16 144 |
| Filter: Response could not be computed | 10 144 | 0 | 13 839 | 0 |
| Filter: Therapy cycle without previous relapse | 10 144 | 0 | 13 839 | 0 |
| Filter: Index therapy DMTs is not one of the 6 study DMTs^b^ | 9417 | -727 | 12 277 | -1562 |
| Filter: Current Therapy is “OtherDMT”, Unknown or not available in the NTD | 9285 | -132 | 12 064 | -213 |
| Filter: More than one therapy cycle per patient (randomly sample one cycle) | 9285 | 0 | 9285 | -2779 |
| Filter: Clinical sites where only one patient is left | 9285 | 0 | 9285 | 0 |
| *EDSS Expanded Disability Status Scale; DMT disease-modifying therapy; MS multiple sclerosis; ARR annualized relapse rate.*  *^a^In the applied quality criteria, “NoDMT” therapy cycle were added to gaps where no therapy was taken. In the derived analysis dataset, a therapy timeline was derived for each patient. This sequentially shows all DMTs that a patient has taken. After this data-processing, a patient could have “NoDMT” as his first therapy cycle with no previous therapy. Since the aim of the model is to aid in choosing an initial therapy or to guide a therapy switch, a patient has to be on a DMT and thus the first therapy cycle was removed.*  *^b^ The 6 study DMTs are: dimethyl fumarate, fingolimod, glatiramer acetate, interferon beta1, natalizumab, and teriflunomide.* | | | | |


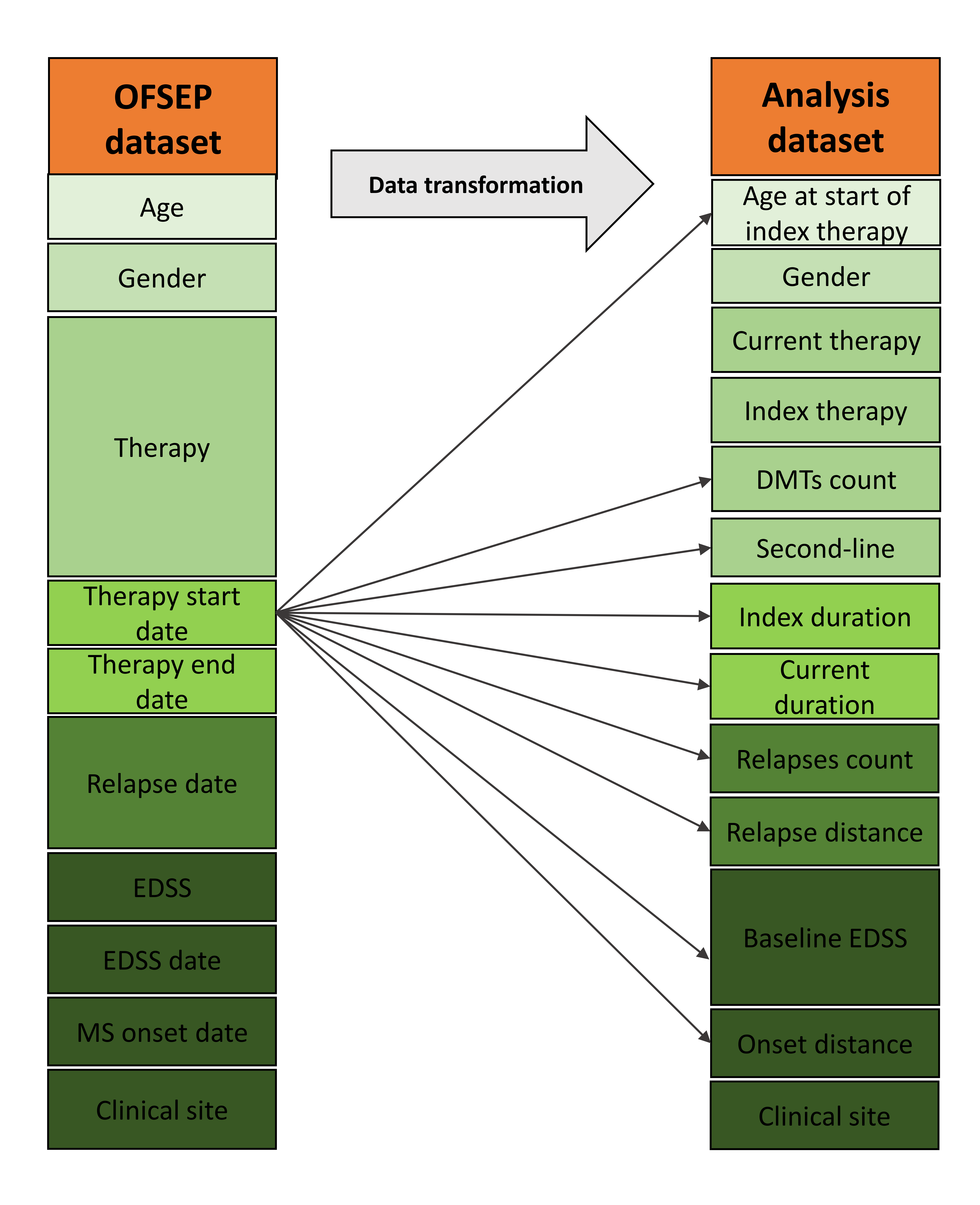


# **Fig.S1** Transformation of the Observatoire Francais de la sclérose en plaque (OFSEP) dataset to derive the predictors.

*MS multiple sclerosis; EDSS Expanded Disability Status Scale; DMT disease-modifying therapy. The predictors indicated by the arrows are defined with respect to the therapy start date. We defined the predictors Index therapy and Baseline EDSS after censoring ongoing therapies on the maximal on-therapy EDSS assessment date occurring before the temporal split date.*

**Table S4** Predictor definitions

| Age | Age in years at the start of the index therapy | Nominal  Less than or equal to 30 (ref), 31 to 40, 41 to 50, 50 or more |
| --- | --- | --- |
| Gender | Gender | Nominal  Female, Male(ref) |
| Baseline EDSS^a^ | Measured at most 6 months before or 3 months after the start of the therapy cycle, and at least 84 days after a relapse | Nominal  1.5 or less (ref), 2 to 2.5, 3 to 3.5, 4 to10; |
| Index therapy | DMT taken during the therapy cycle | Nominal^b^; ref=dimethyl fumarate |
| Index duration | Duration in years of the index therapy | Continuous (year= 365 days).  Its logarithm as offset |
| Current therapy | DMT taken prior to the start of the therapy cycle | Nominal^b^; ref=dimethyl fumarate |
| Current duration | Duration in years of the current therapy | Continuous (year=365 days), z-score by (x-mean(x))/2sd |
| DMTs count | Number of DMTs taken prior to the start of the index therapy | Nominal0, 1, 2, 3 or more(ref) |
| Second-line^c^ | Whether the current therapy or the DMT previous to that was a second-line DMT | Nominal  False (ref), True; |
| Relapse distance | Time elapsed between the last relapse preceding or on the start of the index therapy and the start of the index therapy | Nominal  less than 0.25 (ref), 0.25 to 0.99, 1 to 2.99, 3 or more |
| Relapses count | Number of relapses in the year prior to or on the start of the index therapy | Continuous (year=365 days), z-score by (x-mean(x))/2sd |
| Onset distance | Time elapsed in years between MS onset and start of index therapy | Continuous (year=365 days), z-score by (x-mean(x))/2sd |
| Clinical site | Clinical site where the course of MS is observed | Nominal  As random intercept |
| *EDSS Expanded Disability Status scale; DMT disease-modifying therapy; MS multiple sclerosis, sd standard deviations.^a^If patients had two EDSS measurements with conflicting results on the same day, these measurements were removed. ^b^The categories are: dimethyl fumarate, fingolimod, glatiramer acetate, interferon-ß1, natalizumab, teriflunomide. ^c^The following therapies were considered second-line: alemtuzumab, cyclophosphamide, fingolimod, mitoxantrone, natalizumab, ocrelizumab, rituximab.*  *All reference categories are chosen according to the original article (7) predictors definition.* | | |


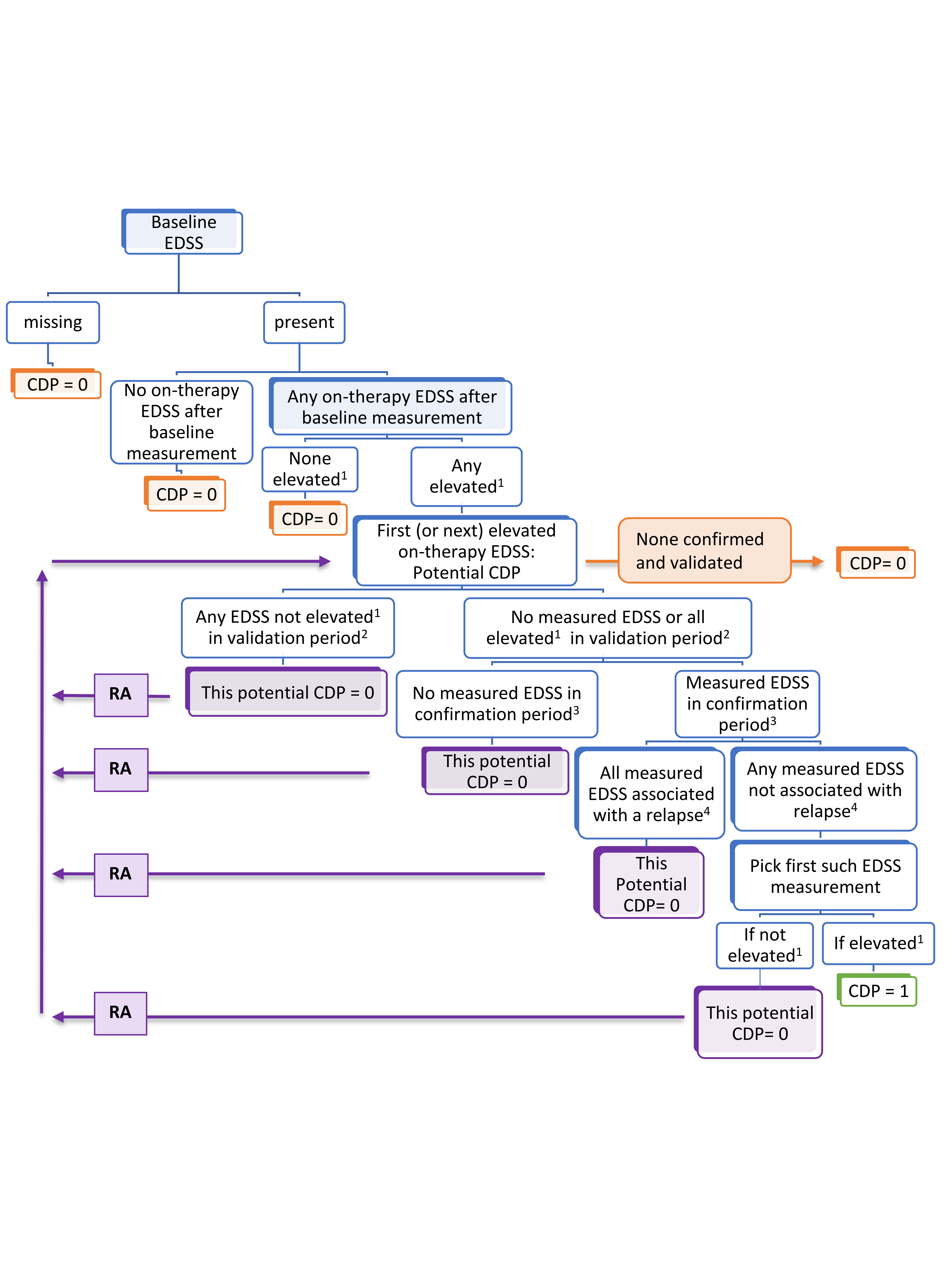


# **Fig.S2** Confirmed disability progression (CDP) definition.

*EDSS Expanded Disability Status scale****,*** *RA: Repeat assessment for all elevated on-therapy EDSS*

*^1^ Elevation: an increase in EDSS by 1 unit if baseline EDSS <= 5.5, by 0.5 unit otherwise*

*^2^ Validation period: > potential CDP date and <= potential CDP date + 84 days*

*^3^ Confirmation period: > potential CDP date + 84 days and <= therapy end date + 365 days*

*^4^ Association to a relapse: relapse date <= EDSS date and <= relapse date + 84 days*

# **Table S5** Required sample size in different scenarios assuming b=1 and for 90% power, alpha=0.05 in a two-sided test.

| **Event Proportion** | **C-index_0_=0.5  C-index_1_=0.55** | **C-index_0_=0.5  C-index_1_=0.6** | **C-index_0_=0.5  C-index_1_=0.65** | **C-index_0_=0.5  C-index_1_=0.7** | **C-index_0_=0.55  C-index_1_=0.6** | **C-index_0_=0.55  C-index_1_=0.65** | **C-index_0_=0.55  C-index_1_=0.7** | **C-index_0_=0.6  C-index_1_=0.65** | **C-index_0_=0.6  C-index_1_=0.7** | **C-index_0_=0.65  C-index_1_=0.7** |
| --- | --- | --- | --- | --- | --- | --- | --- | --- | --- | --- |
| 85% | 2707 | 650 | 270 | 137 | 2596 | 605 | 243 | 2418 | 545 | 2176 |
| 75% | 1843 | 443 | 184 | 94 | 1768 | 412 | 166 | 1647 | 371 | 1483 |
| 50% | 1384 | 334 | 138 | 70 | 1330 | 312 | 126 | 1242 | 280 | 1120 |
| 25% | 1848 | 446 | 186 | 95 | 1779 | 416 | 168 | 1663 | 376 | 1502 |
| 15% | 2719 | 656 | 273 | 139 | 2618 | 613 | 246 | 2449 | 554 | 2213 |
| *A measure equivalent to the C-index is the area under the receiver operating curve (AUC). We used the C-index notation in this section for consistency with the manuscript although our reference for sample size calculation uses the AUC notation (17). In order to be informed about the sample size requirements for different but plausible scenarios, we considered a range of target C-indicess as alternative hypothesis, H_1_ (denoted as C-index_1_) ranging from 0.55 to 0.70 with 0.05 increments. We compared those to a range of minimally acceptable C-indicess as the null hypothesis, H_0_, denoted as C-index_0_, ranging from 0.50 to 0.65 with 0.05 increments. The range of C-indicess are motivated by the range of C-index values reported in Stühler et al (7) for both outcomes. Varying proportions of the population with the event of interest were considered. Assuming a binormal ROC curve with the curvature b=1, we used the C-index values to estimate a in each scenario. Then, the variances of the displacement values were calculated by simulating 10 000 patients with and 10 000 patients without event. For 90% power to reject the null hypothesis at a significance level of alpha=0.05 in a two-sided test, the required sample size ranges between 70 to 2719.* | | | | | | | | | | |

# **Box S1** Model development and diagnostics

The models were estimated using the stan_glmer.nb and stan.glmer functions of the rstanarm R package which is a wrapper around the RStan package. This Bayesian Stan-software-based package uses the Hamiltonian Markov Chain Monte Carlo approach that relies on sampling from the target posterior parameter’s distribution. The MCMC algorithm uses a Markov chain to explore the shape of the estimated posterior distribution (18). The default settings were used, so four chains were started with 2000 iterations each (1000 in each chain was used for warming up) resulting in 4000 draws from each parameter posterior’s distribution. The model diagnostics were performed by investigating its convergence and the sampling quality.

**Model Convergence**

To check whether the algorithm is generating a reliable sample for approximating the posterior distribution, several chains run in parallel because a chain can only be initiated for a limited number of iterations. A convergence of the different chains to the same (equilibrium) distribution indicates a proper approximation of the parameters’ posterior distribution. The convergence of the chains was checked by looking at the R ̂ statistic (the potential scale reduction factor, also known as the Gelman and Rubin statistic R ̂). This statistic is the ratio of the between chain variance to the within chain variance of the different draws. A value close to 1 indicates convergence of the chains to an equilibrium distribution (18). R ̂ was checked for all of our models’ parameters, and all had a value <1.1 indicating convergence.

**Sampling Quality**

Bayesian model estimation via the MCMC algorithm relies on sampling from the target posterior parameter distribution. To make sure the drawn sample is informative and reliable, the below diagnostics were checked for every parameter: the Monte Carlo standard error and the effective sample size.

*Monte Carlo standard error (MCSE)*

The Monte Carlo standard error is the error introduced by the MCMC sampler. It reflects the variation in the sampling error. It should be low relative to the standard deviation of the estimated posterior parameters otherwise it masks the uncertainty of the estimated parameters. This was the case also for all our parameters for both models. The MCSE is inversely related to the effective sample size, where a relatively low MCSE compared to the standard deviation, leads to a higher effective sample size (18).

*Effective sample size (Neff)*

MCMC draws from the target posterior parameter distribution are not independent and the chains will show some levels of auto-correlation. A low level of auto-correlation is desirable because independent draws carry more information about the posterior parameters. The effective sample size is the number of independent draws that can accurately estimate the posterior parameters (18).

All diagnostics are shown alongside model posterior coefficients in tables 10 and 11 for the relapse and CDP models respectively.

# **Table S6** Methodological differences between this study and that of Stühler et al

| **Methods** | **This study** | **Stühler et al** (7) |
| --- | --- | --- |
| Predictor definitions | Onset distance: Time elapsed between MS onset and start of index therapy  Continuous variables standardized with the mean and standard deviation estimated only in the training set. | Diagnosis distance: Time elapsed between MS diagnosis and start of index therapy.  Continuous variables standardized before the data set is split into a training and test sets. |
| Filtration  Criteria | Filtration criteria applied to all data before the temporal split. | Filtration criteria applied separately to training and test sets after the random split. |
|  | Filtered out therapy cycles that started before 01-01-2011 | Filtered out therapy cycles that started before 01-01-2009 |
|  | Filtered out therapy cycles that started within 6 months of MS onset | Filtered out therapy cycles that started within 6 months of MS diagnosis |
| Validation scheme | Data temporally split into training set for model development and test set for model validation.  Performance assessed via 10-fold cross-validation within the training set, and in the test set. | Data randomly split into training set (90%) for model development and test set (10%) for model validation.  Performance assessed via 10-fold cross-validation, leave-one-site-out within the training set, and in the test set. |
| Statistical performance measures | Calculated via the *CalibrationCurves package function val.prob.ci.2* in both validation schemes:  Harrell’s c-index without matching patients on index therapy duration.  Reported C-indices and calibration curves with intercept and slope and their 95% confidence intervals. | Calculated C-indices manually by matching pairs based on their response and disease duration. No report of precision in test set.    Reported calibration visually in cross- validation only, but not in test set. |

***Box S2*** *Session info showing packages used in this study*

R version 4.2.2 Patched (2022-11-10 r83330)

Platform: x86_64-pc-linux-gnu (64-bit)

Running under: Debian GNU/Linux bookworm/sid

Matrix products: default

BLAS: /usr/lib/x86_64-linux-gnu/openblas-pthread/libblas.so.3

LAPACK: /usr/lib/x86_64-linux-gnu/openblas-pthread/libopenblasp-r0.3.21.so

locale:

[1] LC_CTYPE=de_DE.UTF-8 LC_NUMERIC=C

[3] LC_TIME=de_DE.UTF-8 LC_COLLATE=de_DE.UTF-8

[5] LC_MONETARY=de_DE.UTF-8 LC_MESSAGES=de_DE.UTF-8

[7] LC_PAPER=de_DE.UTF-8 LC_NAME=C

[9] LC_ADDRESS=C LC_TELEPHONE=C

[11] LC_MEASUREMENT=de_DE.UTF-8 LC_IDENTIFICATION=C

attached base packages:

[1] stats graphics grDevices utils datasets methods

[7] base

other attached packages:

[1] patchwork_1.1.2 pROC_1.18.0

[3] summarytools_1.0.1 MASS_7.3-58.2

[5] rsample_1.1.1 ggrepel_0.9.3

[7] rstanarm_2.21.3 Rcpp_1.0.10

[9] CalibrationCurves_0.1.5 rms_6.3-0

[11] SparseM_1.81 Hmisc_4.8-0

[13] Formula_1.2-4 survival_3.5-3

[15] lattice_0.20-45 openxlsx_4.2.5.2

[17] lubridate_1.9.1 forcats_1.0.0

[19] stringr_1.5.0 dplyr_1.0.10

[21] purrr_1.0.1 readr_2.1.3

[23] tidyr_1.3.0 tibble_3.1.8

[25] ggplot2_3.4.0 tidyverse_1.3.2

# **Table S7** TRIPOD checklist

| **Section/Topic** | **Item** |  | **Checklist Item** | **Page** |
| --- | --- | --- | --- | --- |
| **Title and abstract** | | | | |
| Title | 1 | D;V | Identify the study as developing and/or validating a multivariable prediction model, the target population, and the outcome to be predicted. | 1 |
| Abstract | 2 | D;V | Provide a summary of objectives, study design, setting, participants, sample size, predictors, outcome, statistical analysis, results, and conclusions. | 2 |
| **Introduction** | | | | |
| Background and objectives | 3a | D;V | Explain the medical context (including whether diagnostic or prognostic) and rationale for developing or validating the multivariable prediction model, including references to existing models. | 4 |
|  | 3b | D;V | Specify the objectives, including whether the study describes the development or validation of the model or both. | 4 |
| **Methods** | | | | |
| Source of data | 4a | D;V | Describe the study design or source of data (e.g., randomized trial, cohort, or registry data), separately for the development and validation data sets, if applicable. | 5 |
|  | 4b | D;V | Specify the key study dates, including start of accrual; end of accrual; and, if applicable, end of follow-up. | 5 |
| Participants | 5a | D;V | Specify key elements of the study setting (e.g., primary care, secondary care, general population) including number and location of centres. | 5 |
|  | 5b | D;V | Describe eligibility criteria for participants. | 5 |
|  | 5c | D;V | Give details of treatments received, if relevant. | 4 |
| Outcome | 6a | D;V | Clearly define the outcome that is predicted by the prediction model, including how and when assessed. | 6 |
|  | 6b | D;V | Report any actions to blind assessment of the outcome to be predicted. | NA |
| Predictors | 7a | D;V | Clearly define all predictors used in developing or validating the multivariable prediction model, including how and when they were measured. | 6 |
|  | 7b | D;V | Report any actions to blind assessment of predictors for the outcome and other predictors. | NA |
| Sample size | 8 | D;V | Explain how the study size was arrived at. | 7 |
| Missing data | 9 | D;V | Describe how missing data were handled (e.g., complete-case analysis, single imputation, multiple imputation) with details of any imputation method. | 7 |
| Statistical analysis methods | 10a | D | Describe how predictors were handled in the analyses. | 8 |
|  | 10b | D | Specify type of model, all model-building procedures (including any predictor selection), and method for internal validation. | 7 |
|  | 10c | V | For validation, describe how the predictions were calculated. | 7, 8 |
|  | 10d | D;V | Specify all measures used to assess model performance and, if relevant, to compare multiple models. | 7, 8 |
|  | 10e | V | Describe any model updating (e.g., recalibration) arising from the validation, if done. | NA |
| Risk groups | 11 | D;V | Provide details on how risk groups were created, if done. | NA |
| Development vs. validation | 12 | V | For validation, identify any differences from the development data in setting, eligibility criteria, outcome, and predictors. | 8 |
| **Results** | | | | |
| Participants | 13a | D;V | Describe the flow of participants through the study, including the number of participants with and without the outcome and, if applicable, a summary of the follow-up time. A diagram may be helpful. | 8, 9 |
|  | 13b | D;V | Describe the characteristics of the participants (basic demographics, clinical features, available predictors), including the number of participants with missing data for predictors and outcome. | 8, 10 |
|  | 13c | V | For validation, show a comparison with the development data of the distribution of important variables (demographics, predictors and outcome). | 8, 10,11 |
| Model development | 14a | D | Specify the number of participants and outcome events in each analysis. | 11 |
|  | 14b | D | If done, report the unadjusted association between each candidate predictor and outcome. | NA |
| Model specification | 15a | D | Present the full prediction model to allow predictions for individuals (i.e., all regression coefficients, and model intercept or baseline survival at a given time point). | 12 |
|  | 15b | D | Explain how to the use the prediction model. | 4 |
| Model performance | 16 | D;V | Report performance measures (with CIs) for the prediction model. | 12,13,14, 15, 16 |
| Model-updating | 17 | V | If done, report the results from any model updating (i.e., model specification, model performance). | NA |
| **Discussion** | | | | |
| Limitations | 18 | D;V | Discuss any limitations of the study (such as nonrepresentative sample, few events per predictor, missing data). | 18 |
| Interpretation | 19a | V | For validation, discuss the results with reference to performance in the development data, and any other validation data. | 17, 18 |
|  | 19b | D;V | Give an overall interpretation of the results, considering objectives, limitations, results from similar studies, and other relevant evidence. | 17, 18 |
| Implications | 20 | D;V | Discuss the potential clinical use of the model and implications for future research. | 18 |
| **Other information** | | | | |
| Supplementary information | 21 | D;V | Provide information about the availability of supplementary resources, such as study protocol, Web calculator, and data sets. | 19 |
| Funding | 22 | D;V | Give the source of funding and the role of the funders for the present study. | 19 |

# **Table S8** Missing data in the transferred and the analysis datasets

| **Data set** | **Missing variable** | **Treatment cycles (%)** |
| --- | --- | --- |
| Transferred | Therapy cycle start date | 835 (1%) |
| Transferred | Therapy cycle end date | 845 (1%) |
| Transferred | EDSS date | 1098 (1%) |
| Transferred | EDSS measurement | 1098 (1%) |
| Analysis | Baseline EDSS | 36507 (38%) |
| Analysis | Relapse distance | 1 (0%) |


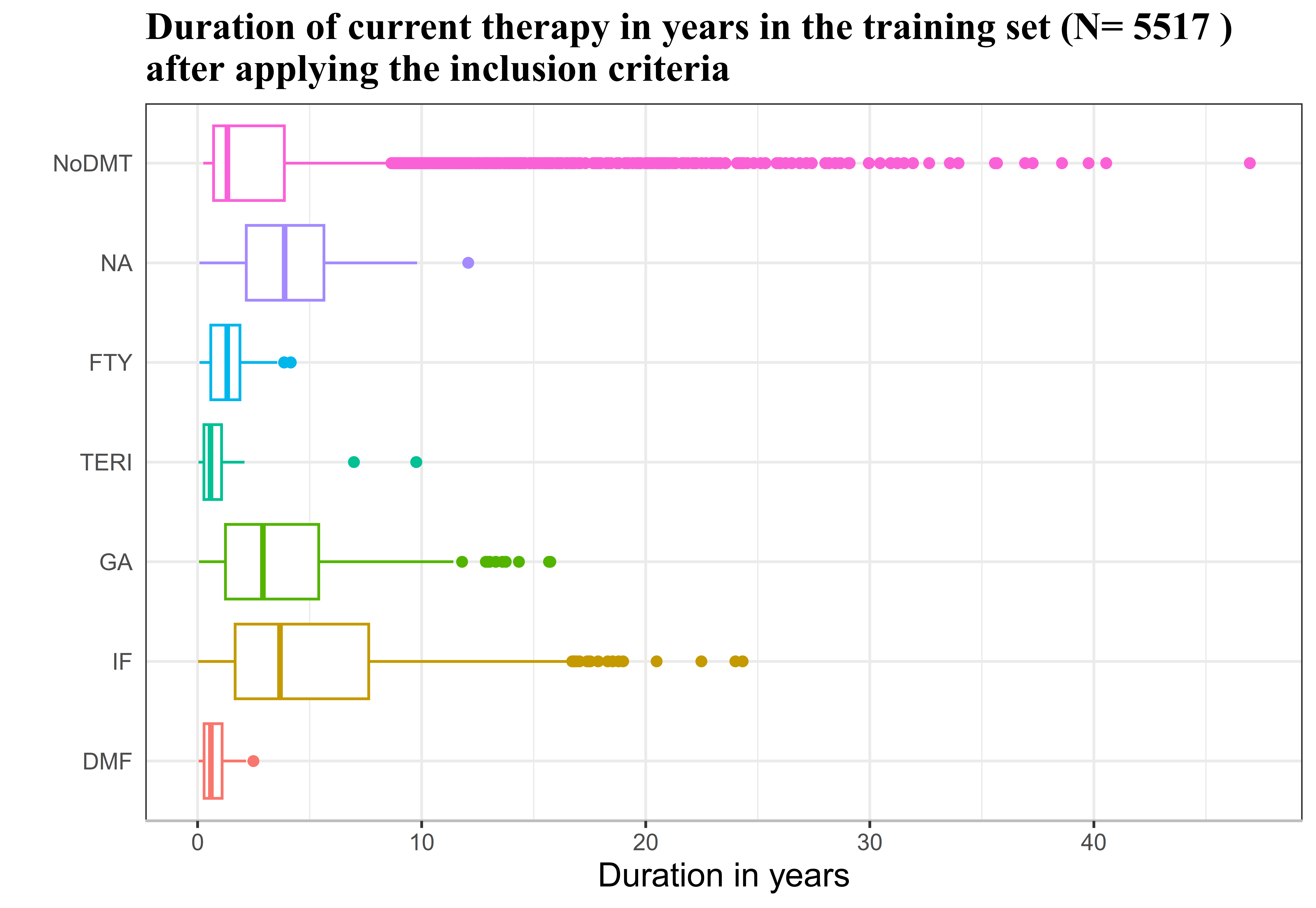


# **Fig.S3** Duration of current therapy in the training set (N=5517).

*NoDMT treatment-free period, NA natalizumab, FTY fingolimod, TERI teriflunomide, GA glatiramer acetate, IF interferon Beta1, DMF dimethyl fumarate.*


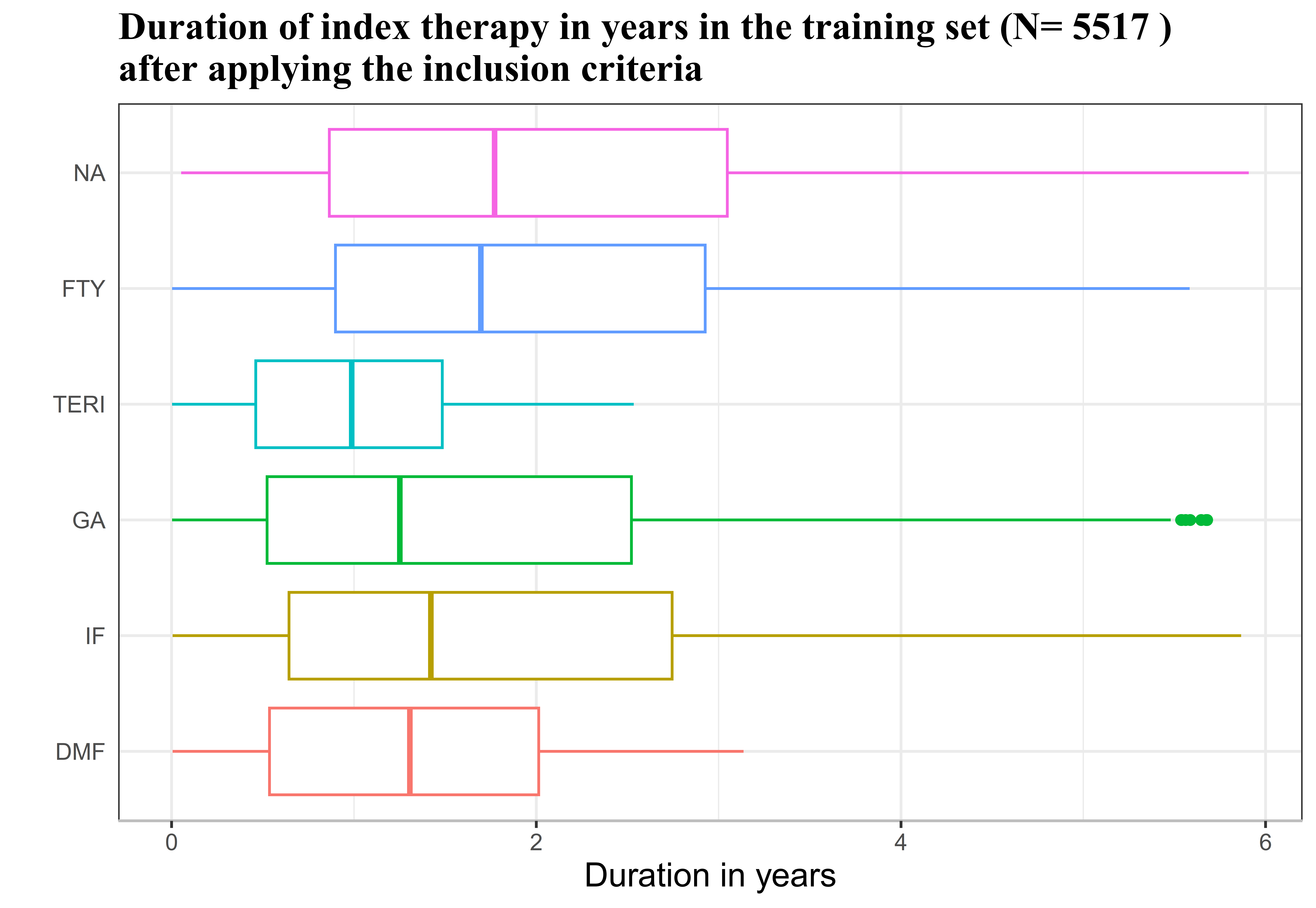


# **Fig.S4** Duration of index therapy in the training set (N=5517).

*NA natalizumab, FTY fingolimod, TERI teriflunomide, GA glatiramer acetate, IF interferon Beta1, DMF dimethyl fumarate.*

# **Table S9** Most important predictors

| **Outcome** | **Rank** | **Predictor^a^** | **Median^b^** | **MAD^c^** |
| --- | --- | --- | --- | --- |
| Relapse | 1 | (Intercept) | -1.808 | 0.441 |
|  | 2 | Age= 50 or more | -0.873 | 0.129 |
|  | 3 | Relapses count | 0.871 | 0.184 |
|  | 4 | Index=NA | -0.765 | 0.282 |
|  | 5 | Current duration | 0.678 | 0.954 |
|  | 6 | Current=FTY:Current duration | 0.628 | 1.632 |
|  | 7 | Current=NoDMT:Current duration | -0.608 | 0.947 |
|  | 8 | Current=FTY | 0.586 | 0.573 |
| CDP | 1 | (Intercept) | -3.399 | 0.641 |
|  | 2 | Current=GA:Current duration | -1.219 | 1.164 |
|  | 3 | EDSS= 3 or 3.5 | -1.163 | 0.182 |
|  | 4 | Current=NoDMT:Current duration | 0.865 | 1.062 |
|  | 5 | EDSS= 4 or more | -0.823 | 0.159 |
|  | 6 | EDSS= 2 or 2.5 | -0.822 | 0.135 |
|  | 7 | Index=GA | 0.793 | 0.359 |
|  | 8 | Index=GA:Gender=Female | -0.775 | 0.401 |
| *TERI teriflunomide; NA natalizumab; FTY fingolimod; IF intereferon Beta1; NoDMT treatment-free period.* ***^a^****Predictors are ranked according to the magnitude of the median of the corresponding coefficients’ posterior distribution;* ***^b^****The medians are computed from 4000 simulations from the posterior coefficient distribution. A positive median is associated with an increase in the number of relapses and vice versa.*  ***^c^****MAD median absolute deviation from the median are computed from the same posterior samples, and are less sensitive to outliers than posterior standard deviations for non-normal distributions.* | | | | |

# **Table S10** Posterior coefficients, 95% credible intervals and diagnostic measures of the relapse model.

| **Predictor** | **Mean^a^** | **MCSE** | **SD** | **2.50%** | **50%** | **97.50%** | **Neff** | **Rhat** | **OFSEP MAD** | **NTD MAD^b^** |
| --- | --- | --- | --- | --- | --- | --- | --- | --- | --- | --- |
| (Intercept) | -1.806 | 0.013 | 0.448 | -2.68 | -1.808 | -0.935 | 1151 | 1.003 | 0.441 | 0.557 |
| 0.25 ≤ Relapse distance < 1 | -0.014 | 0.002 | 0.105 | -0.21 | -0.016 | 0.19 | 3667 | 0.999 | 0.107 | 0.085 |
| 1 ≤ Relapse distance < 3 | -0.016 | 0.003 | 0.159 | -0.326 | -0.016 | 0.301 | 3002 | 1 | 0.155 | 0.164 |
| Relapse distance ≥ 3 | -0.364 | 0.003 | 0.169 | -0.696 | -0.364 | -0.03 | 3045 | 1 | 0.164 | 0.164 |
| 1.5 < EDSS ≤ 2.5 | 0.294 | 0.001 | 0.08 | 0.136 | 0.294 | 0.452 | 4876 | 1.001 | 0.082 | 0.087 |
| 2.5 < EDSS ≤ 3.5 | 0.309 | 0.002 | 0.097 | 0.117 | 0.311 | 0.499 | 4096 | 1.001 | 0.097 | 0.11 |
| 3.5 < EDSS ≤ 10 | 0.437 | 0.001 | 0.092 | 0.257 | 0.437 | 0.616 | 4235 | 1 | 0.093 | 0.109 |
| Second line=TRUE | 0.07 | 0.005 | 0.238 | -0.407 | 0.076 | 0.537 | 2280 | 1.001 | 0.243 | 0.288 |
| DMTs count=1 | 0.495 | 0.002 | 0.113 | 0.279 | 0.494 | 0.711 | 3249 | 0.999 | 0.115 | 0.114 |
| DMTs count=2 | 0.469 | 0.002 | 0.133 | 0.206 | 0.468 | 0.727 | 3368 | 1 | 0.136 | 0.147 |
| DMTs count=3 or more | 0.524 | 0.003 | 0.148 | 0.234 | 0.526 | 0.817 | 3494 | 0.999 | 0.147 | 0.175 |
| Relapses count | 0.874 | 0.004 | 0.185 | 0.519 | 0.871 | 1.251 | 2276 | 1.003 | 0.184 | 0.234 |
| Onset distance | -0.042 | 0.004 | 0.179 | -0.395 | -0.038 | 0.3 | 1798 | 1 | 0.185 | 0.201 |
| Gender=Female | 0.146 | 0.005 | 0.182 | -0.215 | 0.145 | 0.508 | 1567 | 1.003 | 0.184 | 0.196 |
| 30 < Age ≤ 40 | -0.104 | 0.001 | 0.085 | -0.274 | -0.104 | 0.067 | 4186 | 1.001 | 0.082 | 0.09 |
| 40 < Age ≤ 50 | -0.386 | 0.002 | 0.095 | -0.572 | -0.385 | -0.197 | 3481 | 1.001 | 0.097 | 0.089 |
| Age > 50 | -0.872 | 0.002 | 0.125 | -1.114 | -0.873 | -0.63 | 3847 | 1.002 | 0.129 | 0.117 |
| Current duration | 0.668 | 0.03 | 0.943 | -1.211 | 0.678 | 2.52 | 982 | 1.002 | 0.954 | 1.41 |
| Current=IF | -0.351 | 0.012 | 0.376 | -1.092 | -0.356 | 0.372 | 1029 | 1.002 | 0.385 | 0.494 |
| Current=GA | -0.244 | 0.012 | 0.378 | -0.985 | -0.248 | 0.497 | 1032 | 1.001 | 0.379 | 0.489 |
| Current=TERI | 0.219 | 0.012 | 0.69 | -1.212 | 0.234 | 1.541 | 3120 | 1 | 0.675 | 1.406 |
| Current=FTY | 0.578 | 0.012 | 0.557 | -0.531 | 0.586 | 1.643 | 2339 | 1 | 0.573 | 0.615 |
| Current=NA | 0.157 | 0.012 | 0.391 | -0.612 | 0.155 | 0.921 | 1083 | 1.001 | 0.39 | 0.521 |
| Current=NoDMT | -0.178 | 0.012 | 0.375 | -0.919 | -0.186 | 0.559 | 1038 | 1.001 | 0.374 | 0.492 |
| Index=IF | 0.303 | 0.005 | 0.233 | -0.141 | 0.307 | 0.754 | 1995 | 1.002 | 0.24 | 0.269 |
| Index=GA | 0.328 | 0.005 | 0.241 | -0.148 | 0.328 | 0.805 | 1950 | 1.001 | 0.232 | 0.304 |
| Index=TERI | 0.282 | 0.005 | 0.234 | -0.165 | 0.28 | 0.747 | 2195 | 1.001 | 0.239 | 0.267 |
| Index=FTY | -0.291 | 0.005 | 0.2 | -0.678 | -0.29 | 0.101 | 1760 | 1.002 | 0.204 | 0.242 |
| Index=NA | -0.761 | 0.006 | 0.285 | -1.337 | -0.765 | -0.212 | 2234 | 1.002 | 0.282 | 0.354 |
| Index=IF:Relapses count | -0.217 | 0.004 | 0.203 | -0.626 | -0.217 | 0.188 | 2657 | 1.002 | 0.197 | 0.216 |
| Index=GA:Relapses count | -0.446 | 0.004 | 0.216 | -0.876 | -0.445 | -0.022 | 3010 | 1.002 | 0.215 | 0.245 |
| Index=TERI:Relapses count | -0.483 | 0.005 | 0.271 | -1.038 | -0.477 | 0.051 | 3450 | 1.002 | 0.268 | 0.3 |
| Index=FTY:Relapses count | -0.269 | 0.004 | 0.185 | -0.646 | -0.271 | 0.095 | 2427 | 1.002 | 0.186 | 0.189 |
| Index=NA:Relapses count | -0.466 | 0.004 | 0.225 | -0.895 | -0.465 | -0.03 | 2777 | 1.003 | 0.224 | 0.287 |
| Index=IF:Second line=TRUE | 0.246 | 0.006 | 0.33 | -0.398 | 0.246 | 0.884 | 3025 | 1 | 0.326 | 0.334 |
| Index=GA:Second-line=TRUE | 0.329 | 0.006 | 0.316 | -0.275 | 0.318 | 0.967 | 2891 | 1 | 0.314 | 0.378 |
| Index=TERI:Second line=TRUE | 0.165 | 0.007 | 0.406 | -0.609 | 0.157 | 0.969 | 3649 | 1 | 0.408 | 0.418 |
| Index=FTY:Second line=TRUE | 0.387 | 0.005 | 0.243 | -0.082 | 0.381 | 0.875 | 2531 | 1 | 0.241 | 0.307 |
| Index=NA:Second line=TRUE | -0.055 | 0.006 | 0.321 | -0.691 | -0.046 | 0.561 | 3171 | 0.999 | 0.314 | 0.405 |
| Index=IF:Gender=Female | 0.09 | 0.006 | 0.245 | -0.376 | 0.089 | 0.578 | 1925 | 1.002 | 0.251 | 0.244 |
| Index=GA:Gender=Female | -0.081 | 0.006 | 0.259 | -0.583 | -0.078 | 0.43 | 2012 | 1.001 | 0.258 | 0.296 |
| Index=TERI:Gender=Female | -0.09 | 0.005 | 0.265 | -0.612 | -0.087 | 0.418 | 2349 | 1.001 | 0.266 | 0.271 |
| Index=FTY:Gender=Female | 0.097 | 0.005 | 0.218 | -0.318 | 0.096 | 0.528 | 1741 | 1.002 | 0.222 | 0.24 |
| Index=NA:Gender=Female | 0.197 | 0.006 | 0.302 | -0.39 | 0.19 | 0.793 | 2193 | 1.002 | 0.305 | 0.352 |
| Index=IF:Onset distance | -0.347 | 0.005 | 0.249 | -0.842 | -0.345 | 0.143 | 2849 | 1 | 0.249 | 0.248 |
| Index=GA:Onset distance | -0.303 | 0.005 | 0.268 | -0.82 | -0.3 | 0.228 | 3213 | 1 | 0.262 | 0.3 |
| Index=TERI:Onset distance | -0.213 | 0.005 | 0.243 | -0.688 | -0.209 | 0.252 | 2744 | 1 | 0.25 | 0.246 |
| Index=FTY:Onset distance | 0.073 | 0.004 | 0.205 | -0.331 | 0.072 | 0.463 | 2128 | 1 | 0.206 | 0.215 |
| Index=NA:Onset distance | 0.209 | 0.005 | 0.269 | -0.303 | 0.208 | 0.739 | 3262 | 0.999 | 0.27 | 0.333 |
| Current=IF:Current duration | -0.411 | 0.03 | 0.951 | -2.245 | -0.422 | 1.496 | 985 | 1.002 | 0.952 | 1.361 |
| Current=GA:Current duration | -0.285 | 0.03 | 0.971 | -2.16 | -0.28 | 1.642 | 1031 | 1.001 | 0.973 | 1.391 |
| Current=TERI:Current duration | 0.532 | 0.029 | 1.734 | -2.98 | 0.572 | 3.858 | 3483 | 1 | 1.74 | 4.273 |
| Current=FTY:Current duration | 0.603 | 0.03 | 1.645 | -2.634 | 0.628 | 3.764 | 2950 | 1 | 1.632 | 1.968 |
| Current=NA:Current duration | -0.013 | 0.031 | 1.006 | -1.977 | -0.019 | 1.98 | 1085 | 1.002 | 1.003 | 1.618 |
| Current=NoDMT:Current duration | -0.604 | 0.03 | 0.948 | -2.465 | -0.608 | 1.265 | 985 | 1.002 | 0.947 | 1.394 |
| *OFSEP Observatoire Francais de la sclérose en plaque; NTD NeuroTransData; MCSE Monte Carlo standard error; SD standard deviation, Neff effective sample size; Rhat potential scale reduction statistic R; MAD median absolute deviation from median; IF interferon beta1; GA glatiramer acetate; TERI teriflunomide; FTY fingolimod; NA natalizumab; NoDMT treatment-free period.* ***^a^****Mean of the simulations from the posterior parameters distributions.*  ***^b^****MADs extracted from the publication of the original article (7) by means of the PlotDigitizer data extraction tool.* | | | | | | | | | | |

# **Table S11** Posterior coefficients, 95% credible intervals and diagnostic measures of the CDP model

| **Predictor** | **Mean^a^** | **MCSE** | **SD** | **2.50%** | **50%** | **97.50%** | **Neff** | **Rhat** | **OFSEP MAD** | **NTD**  **MAD*^b^*** |
| --- | --- | --- | --- | --- | --- | --- | --- | --- | --- | --- |
| (Intercept) | -3.393 | 0.02 | 0.624 | -4.64 | -3.399 | -2.199 | 999 | 1.003 | 0.641 | 0.731 |
| 0.25 ≤ Relapse distance < 1 | 0.247 | 0.004 | 0.214 | -0.173 | 0.249 | 0.685 | 2362 | 1 | 0.215 | 0.2 |
| 1 ≤ Relapse distance < 3 | 0.211 | 0.007 | 0.306 | -0.362 | 0.199 | 0.804 | 1828 | 1 | 0.306 | 0.299 |
| Relapse distance ≥ 3 | -0.006 | 0.007 | 0.316 | -0.607 | -0.013 | 0.629 | 1908 | 1 | 0.31 | 0.306 |
| 1.5 < EDSS ≤ 2.5 | -0.827 | 0.002 | 0.138 | -1.096 | -0.822 | -0.557 | 4555 | 1 | 0.135 | 0.171 |
| 2.5 < EDSS ≤ 3.5 | -1.167 | 0.003 | 0.183 | -1.53 | -1.163 | -0.812 | 4828 | 1 | 0.182 | 0.199 |
| 3.5 < EDSS ≤ 10 | -0.822 | 0.002 | 0.158 | -1.138 | -0.823 | -0.516 | 4553 | 0.999 | 0.159 | 0.2 |
| Second line=TRUE | 0.339 | 0.009 | 0.382 | -0.433 | 0.347 | 1.034 | 1765 | 1.001 | 0.379 | 0.476 |
| DMTs count=1 | 0.06 | 0.004 | 0.203 | -0.336 | 0.061 | 0.464 | 2307 | 1.001 | 0.202 | 0.223 |
| DMTs count=2 | -0.023 | 0.005 | 0.238 | -0.49 | -0.022 | 0.44 | 2276 | 1.001 | 0.24 | 0.273 |
| DMTs count=3 or more | 0.267 | 0.006 | 0.266 | -0.25 | 0.266 | 0.784 | 2322 | 1.001 | 0.261 | 0.329 |
| Relapses count | 0.14 | 0.008 | 0.339 | -0.551 | 0.146 | 0.784 | 1636 | 1.001 | 0.341 | 0.389 |
| Onset distance | -0.118 | 0.007 | 0.287 | -0.695 | -0.118 | 0.427 | 1722 | 1 | 0.287 | 0.326 |
| Gender=Female | 0.176 | 0.008 | 0.278 | -0.36 | 0.169 | 0.751 | 1168 | 1.002 | 0.275 | 0.297 |
| 30 < Age ≤ 40 | 0.105 | 0.003 | 0.151 | -0.187 | 0.104 | 0.404 | 2930 | 1.001 | 0.149 | 0.184 |
| 40 < Age ≤ 50 | 0.428 | 0.003 | 0.159 | 0.12 | 0.432 | 0.73 | 2706 | 1.002 | 0.162 | 0.185 |
| Age > 50 | 0.556 | 0.004 | 0.19 | 0.175 | 0.557 | 0.931 | 2683 | 1.001 | 0.185 | 0.22 |
| Current duration | -0.738 | 0.035 | 1.041 | -2.824 | -0.729 | 1.292 | 873 | 1.006 | 1.04 | 1.609 |
| Current=IF | 0.161 | 0.016 | 0.495 | -0.777 | 0.149 | 1.172 | 948 | 1.004 | 0.481 | 0.632 |
| Current=GA | 0.02 | 0.016 | 0.503 | -0.935 | 0.014 | 1.03 | 945 | 1.004 | 0.505 | 0.652 |
| Current=TERI | -0.149 | 0.017 | 0.98 | -2.194 | -0.129 | 1.697 | 3261 | 1 | 0.972 | 1.462 |
| Current=FTY | 0.225 | 0.015 | 0.769 | -1.346 | 0.235 | 1.688 | 2502 | 1 | 0.75 | 0.958 |
| Current=NA | 0.343 | 0.017 | 0.529 | -0.674 | 0.333 | 1.407 | 992 | 1.003 | 0.525 | 0.737 |
| Current=NoDMT | 0.27 | 0.016 | 0.489 | -0.677 | 0.264 | 1.272 | 919 | 1.004 | 0.479 | 0.627 |
| Index=IF | 0.216 | 0.01 | 0.375 | -0.536 | 0.213 | 0.94 | 1476 | 1 | 0.37 | 0.343 |
| Index=GA | 0.795 | 0.009 | 0.363 | 0.091 | 0.793 | 1.522 | 1566 | 1 | 0.359 | 0.419 |
| Index=TERI | -0.722 | 0.011 | 0.459 | -1.687 | -0.714 | 0.141 | 1854 | 1.001 | 0.453 | 0.352 |
| Index=FTY | 0.453 | 0.008 | 0.313 | -0.146 | 0.446 | 1.1 | 1420 | 1 | 0.309 | 0.321 |
| Index=NA | 0.173 | 0.01 | 0.424 | -0.628 | 0.162 | 1 | 1794 | 1.001 | 0.421 | 0.455 |
| Index=IF:Relapses count | 0.212 | 0.008 | 0.368 | -0.507 | 0.215 | 0.93 | 2308 | 1.001 | 0.374 | 0.442 |
| Index=GA:Relapses count | -0.329 | 0.008 | 0.388 | -1.086 | -0.329 | 0.426 | 2359 | 1 | 0.387 | 0.518 |
| Index=TERI:Relapses count | -0.236 | 0.009 | 0.527 | -1.283 | -0.226 | 0.78 | 3459 | 1 | 0.528 | 0.555 |
| Index=FTY:Relapses count | -0.264 | 0.008 | 0.329 | -0.902 | -0.27 | 0.386 | 1875 | 1.001 | 0.332 | 0.406 |
| Index=NA:Relapses count | -0.185 | 0.008 | 0.38 | -0.923 | -0.191 | 0.547 | 2135 | 1 | 0.382 | 0.52 |
| Index=IF:Second line=TRUE | 0.349 | 0.011 | 0.554 | -0.742 | 0.337 | 1.429 | 2530 | 1.001 | 0.556 | 0.654 |
| Index=GA:Second line=TRUE | 0.044 | 0.011 | 0.552 | -1.034 | 0.043 | 1.119 | 2659 | 1.001 | 0.558 | 0.674 |
| Index=TERI:Second line=TRUE | -0.426 | 0.015 | 0.839 | -2.219 | -0.368 | 1.064 | 3310 | 1.001 | 0.814 | 1.024 |
| Index=FTY:Second line=TRUE | -0.058 | 0.009 | 0.392 | -0.83 | -0.071 | 0.726 | 2118 | 1.001 | 0.39 | 0.534 |
| Index=NA:Second line=TRUE | -0.034 | 0.01 | 0.497 | -1.004 | -0.027 | 0.915 | 2329 | 1.001 | 0.488 | 0.877 |
| Index=IF:Gender=Female | -0.354 | 0.011 | 0.399 | -1.113 | -0.352 | 0.453 | 1396 | 1.002 | 0.392 | 0.428 |
| Index=GA:Gender=Female | -0.78 | 0.01 | 0.395 | -1.56 | -0.775 | 0.001 | 1600 | 1 | 0.401 | 0.483 |
| Index=TERI:Gender=Female | 0.53 | 0.011 | 0.501 | -0.43 | 0.529 | 1.541 | 2018 | 1.001 | 0.492 | 0.457 |
| Index=FTY:Gender=Female | -0.211 | 0.009 | 0.33 | -0.861 | -0.203 | 0.417 | 1365 | 1.001 | 0.328 | 0.399 |
| Index=NA:Gender=Female | 0.163 | 0.011 | 0.443 | -0.712 | 0.159 | 1.037 | 1751 | 1.001 | 0.436 | 0.547 |
| Index=IF:Onset distance | 0.075 | 0.008 | 0.384 | -0.672 | 0.074 | 0.823 | 2445 | 1 | 0.395 | 0.424 |
| Index=GA:Onset distance | -0.322 | 0.008 | 0.438 | -1.193 | -0.317 | 0.514 | 2940 | 1 | 0.442 | 0.535 |
| Index=TERI:Onset distance | -0.439 | 0.009 | 0.441 | -1.318 | -0.429 | 0.425 | 2519 | 1 | 0.439 | 0.454 |
| Index=FTY:Onset distance | 0.111 | 0.007 | 0.329 | -0.553 | 0.115 | 0.751 | 2195 | 1 | 0.323 | 0.402 |
| Index=NA:Onset distance | -0.092 | 0.008 | 0.421 | -0.937 | -0.086 | 0.722 | 2710 | 1 | 0.409 | 0.564 |
| Current=IF:Current duration | 0.733 | 0.035 | 1.053 | -1.346 | 0.709 | 2.853 | 895 | 1.005 | 1.048 | 1.644 |
| Current=GA:Current duration | -1.204 | 0.036 | 1.166 | -3.472 | -1.219 | 1.157 | 1058 | 1.004 | 1.164 | 1.671 |
| Current=TERI:Current duration | -0.472 | 0.035 | 2.197 | -4.89 | -0.466 | 3.817 | 3908 | 1 | 2.205 | 5.012 |
| Current=FTY:Current duration | -0.543 | 0.034 | 2.002 | -4.601 | -0.533 | 3.322 | 3381 | 1 | 1.963 | 3.089 |
| Current=NA:Current duration | 0.693 | 0.035 | 1.136 | -1.515 | 0.677 | 2.993 | 1038 | 1.005 | 1.101 | 2.152 |
| Current=NoDMT:Current duration | 0.866 | 0.035 | 1.047 | -1.192 | 0.865 | 2.95 | 885 | 1.006 | 1.062 | 1.645 |
| *CDP confimred disease progression; OFSEP Observatoire Francais de la sclérose en plaque; NTD NeuroTransData; MCSE Monte Carlo standard error; SD standard deviation, N_eff effective sample size; Rhat potential scale reduction statistic R; MAD median absolute deviation; IF interferon beta1; GA glatiramer acetate; TERI teriflunomide; FTY fingolimod; NA natalizumab; NoDMT treatment-free period.* ***^a^****Mean of the simulations from the posterior parameter distributions.*  ***^b^****MADs extracted from the publication of the original article (7) by means of the PlotDigitizer data extraction tool.* | | | | | | | | | | |

***Box S3****Model calibration*

The TRIPOD guideline (20) recommends assessing the calibration of prediction models before they are used in clinical practice. This is especially important when such models are intended to guide clinical decisions. A prediction model with high discrimination but bad calibration can be misleading and clinically irrelevant, possibly leading to over-treatment or under-treatment decisions. On the opposite, a model with good calibration and average discrimination could be clinically useful (11).

In our study, we assessed the agreement between the predicted risks and observed proportions using two levels of calibration:

1- “Weak calibration” by fitting a logistic regression model and estimating an intercept (calibration-in-the-large, ideally 0) and slope (quantifying spread of the risk estimates, ideally 1). This level shows on average over- or under-predictions and whether a model have extreme outcome predictions (i.e. high predicted risks for those with the outcome and low predicted risks for those without the outcome).

2- “Moderate calibration” by fitting a flexible calibration curve (loess) to show if the predicted risks of the outcome agree with the observed proportions. This estimation was possible given our large sample size and the large number of events and non-events for both studied outcomes.

“Moderate calibration” is a higher level of calibration because it can detect non-linear relationships (miscalibration) between observed and predicted risks, despite good “weak calibration” (good intercept and slope). For example, in internal validation, if non-linear predictors or interaction terms are poorly modeled, it could result in good calibration intercept and slope but a calibration curve that is not aligned with the diagonal.

Figures 7 and 8 illustrate the two calibration levels mentioned above.


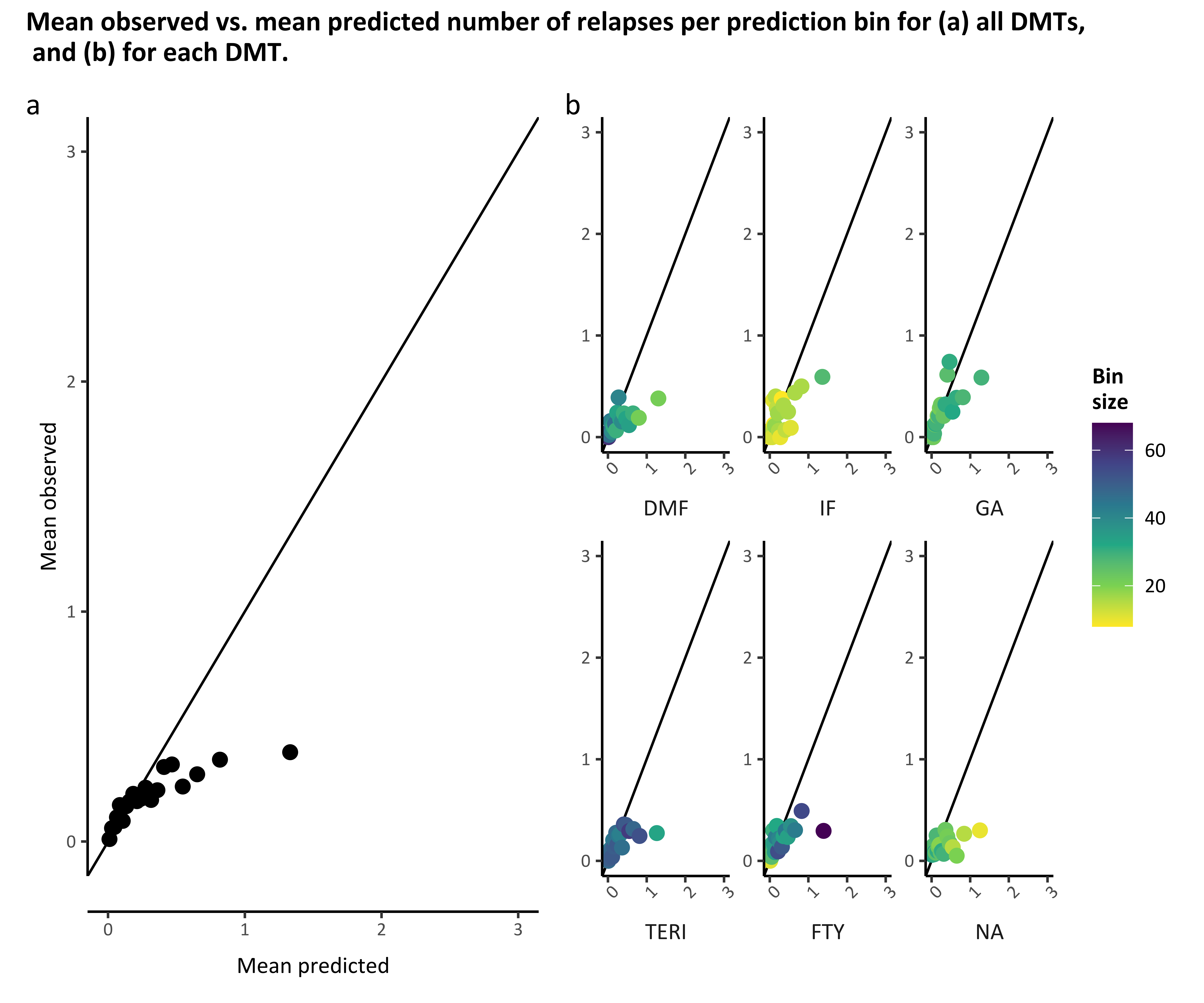


# **Fig.S5** Calibration plot of the relapse model in the test set.

*Shown is the mean observed versus mean predicted number of relapses per prediction bin: panel a: Overall (N= 188 or 189 patients/bin) and panel b: for each DMT. DMF dimethyl fumarate; FTY fingolimod; GA glatiramer acetate; IF interferon beta1; NA natalizumab; TERI teriflunomide.*


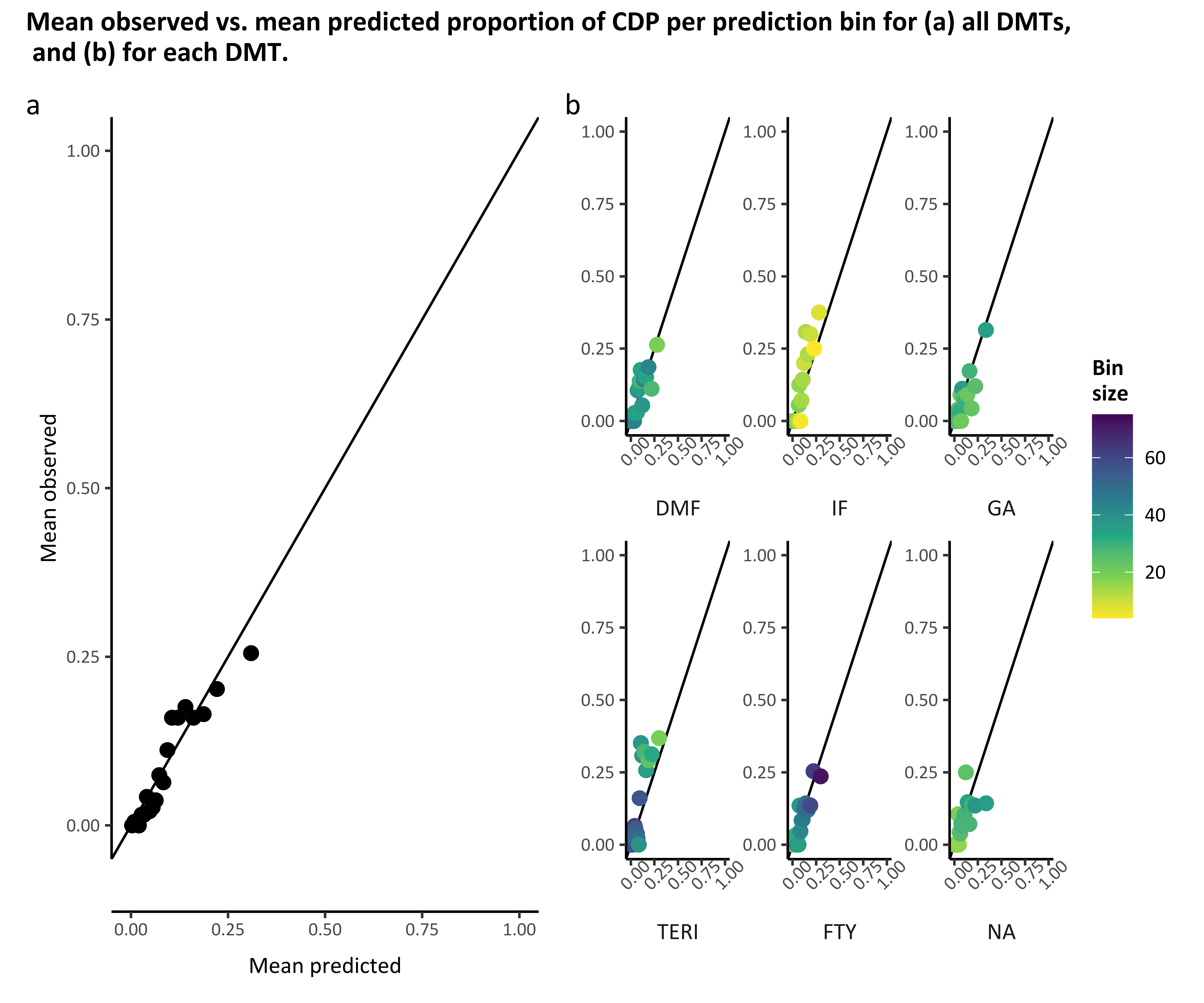


# **Fig.S6** Calibration plot of the confirmed disease progression (CDP) model in the test set**.**

*Shown is the mean observed versus mean predicted proportion of CDP per prediction bin: panel a: Overall (N= 188 or 189 patients/bin) and panel b: for each DMT. DMF dimethyl fumarate; FTY fingolimod; GA glatiramer acetate; IF interferon beta1; NA natalizumab; TERI teriflunomide.*


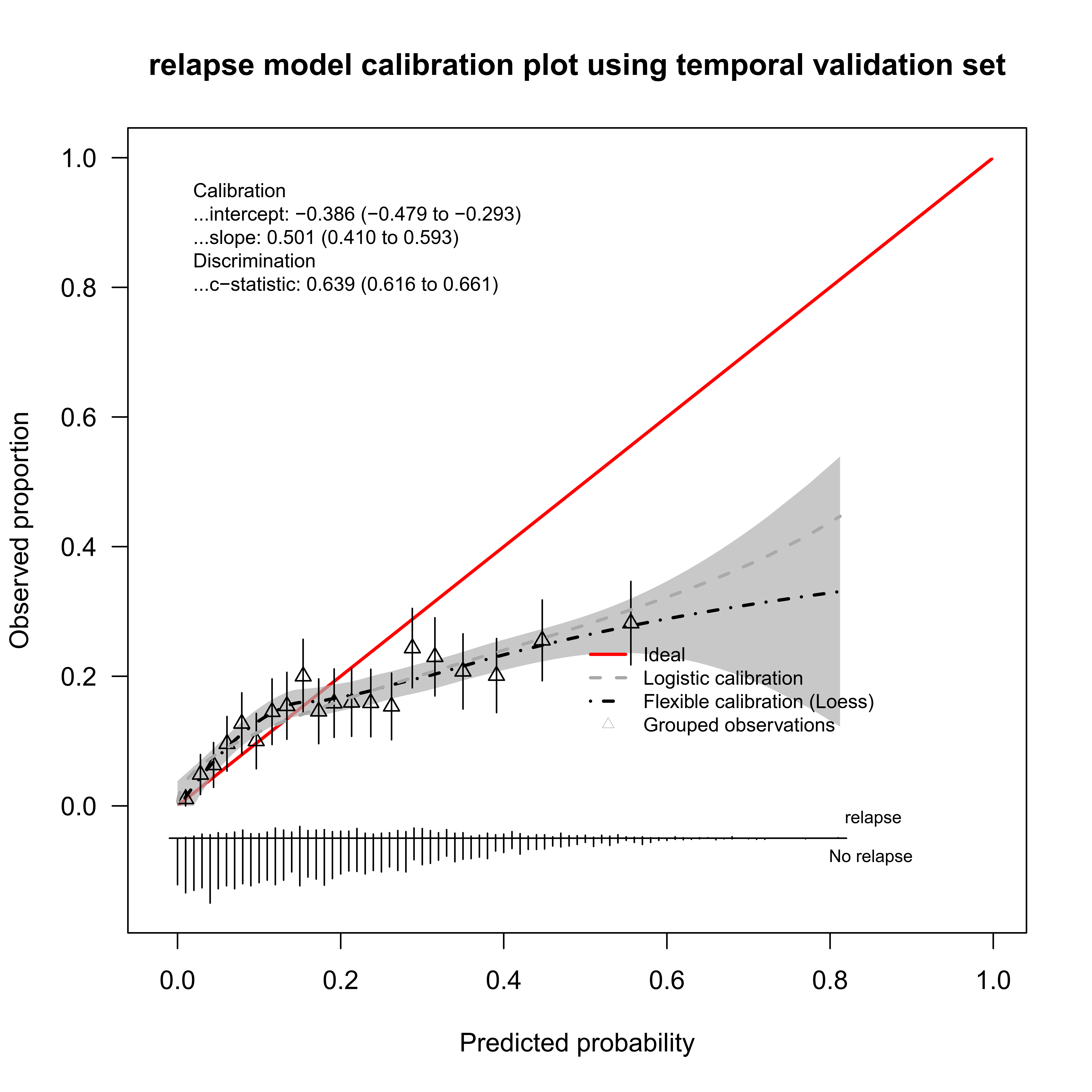


# **Fig.S7** Loess calibration plot of the relapse model in the test set. Observations are divided into 20 groups by their observed relapse.


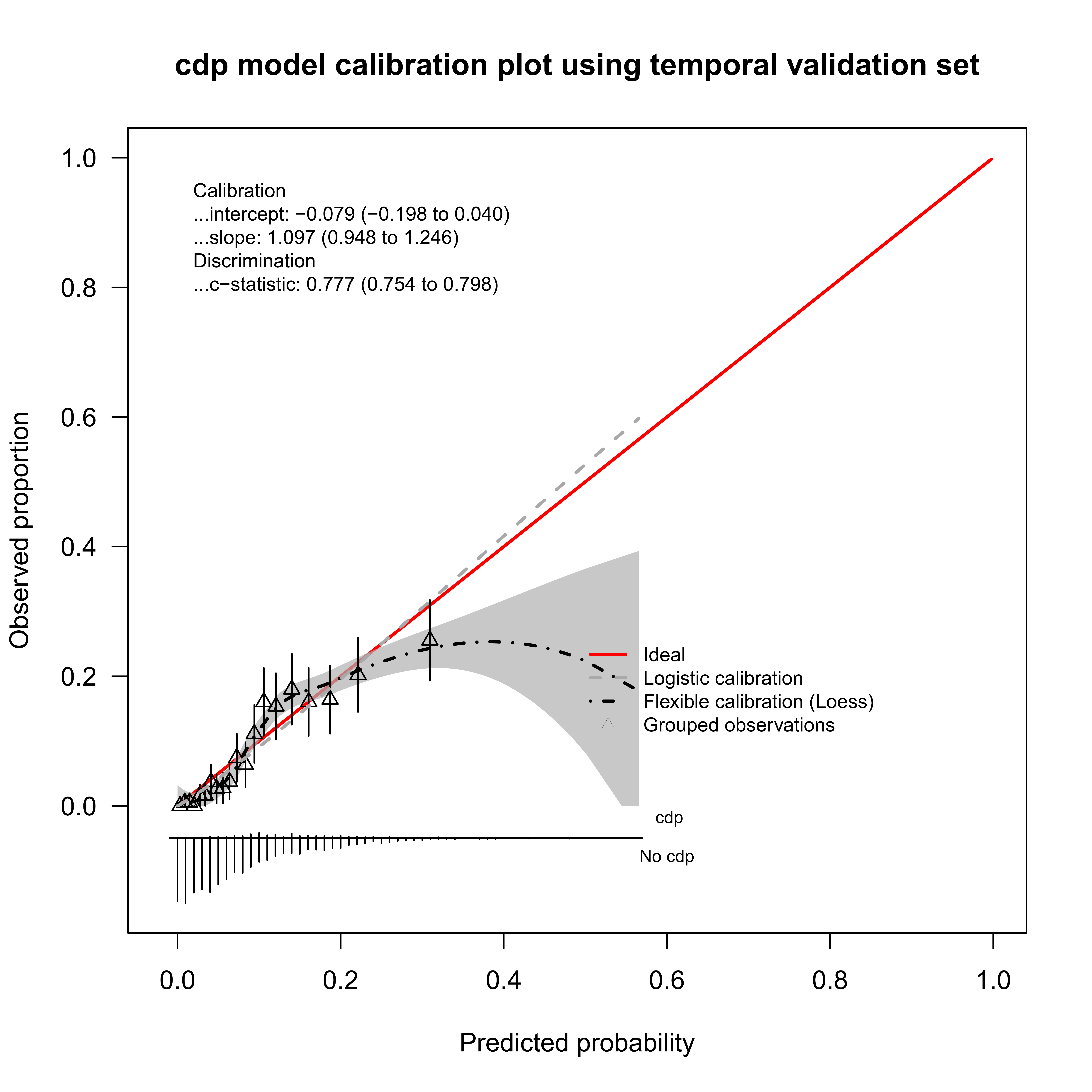


# **Fig.S8** Loess calibration plot of the confirmed disease progression (CDP) model in the test set. Observations are divided into 20 groups by their observed CDP.

# **Table S12** Overview of predictors at baseline in the sensitivity analysis of splitting the dataset in 2019.

| **Predictor** | **Category** | **Training set (N= 8159)**  median (interquartile range; range) or count (%) | **Test set (N= 1236)**  median (interquartile range; range) or count (%) |
| --- | --- | --- | --- |
| Current therapy duration^a^ |  | 1.92 (4.04; 0.01-46.96) | 1.66 (3.4; 0.04-42.07) |
| Index therapy duration |  | 1.74 (2.65; 0-8.47) | 0.77 (0.82; 0.01-2.32) |
| Onset distance^b^ |  | 7.05 (10.24; 0.5-54.96) | 5.05 (9.32; 0.5-42.08) |
| Relapses count |  | 0 (1; 0-5) | 0 (1; 0-3) |
| Age | Less than 30 | 1415 (17) | 245 (20) |
|  | 31 to 40 | 2751 (34) | 493 (40) |
|  | 41 to 50 | 2449 (30) | 287 (23) |
|  | 51 or older | 1544 (19) | 211 (17) |
| Current therapy | dimethylfumarate | 351 (4) | 121 (10) |
|  | fingolimod | 159 (2) | 56 (5) |
|  | glatirameracetate | 759 (9) | 91 (7) |
|  | interferon beta1 | 1633 (20) | 115 (9) |
|  | natalizumab | 654 (8) | 20 (2) |
|  | teriflunomide | 261 (3) | 92 (7) |
|  | NoDMT | 4342 (53) | 741 (60) |
| DMTs count | 0 | 2205 (27) | 406 (33) |
|  | 1 | 3035 (37) | 454 (37) |
|  | 2 | 1663 (20) | 222 (18) |
|  | 3 or more | 1256 (15) | 154 (12) |
| baseline EDSS | 1.5 or less | 3733 (46) | 708 (57) |
|  | 2 to 2.5 | 1982 (24) | 296 (24) |
|  | 3 to 3.5 | 1087 (13) | 132 (11) |
|  | 4 to 10 | 1357 (17) | 100 (8) |
| Female |  | 6137 (75) | 963 (78) |
| Index therapy | dimethyl fumarate | 1560 (19) | 267 (22) |
|  | fingolimod | 1935 (24) | 225 (18) |
|  | glatiramer acetate | 1009 (12) | 166 (13) |
|  | interferon beta1 | 1134 (14) | 93 (8) |
|  | natalizumab | 849 (10) | 188 (15) |
|  | teriflunomide | 1672 (20) | 297 (24) |
| Relapse distance | less than 0.25 | 592 (7) | 59 (5) |
|  | 0.25 to 0.99 | 2787 (34) | 365 (30) |
|  | 1 to 2.99 | 2411 (30) | 385 (31) |
|  | 3 or more | 2369 (29) | 427 (35) |
| Second-line therapy^c^ |  | 1441 (18) | 149 (12) |
| *DMT disease-modifying therapy; EDSS Expanded Disability Status Scale; Continuous variables (in years) summarized by median (interquartile range; range); categorical variables by count (%); ^a^The high maximum duration of current therapy is due to therapy-free periods (NoDMT) before the index therapy. ^b^Onset distance in this study is based on disease onset compared to Diagnosis distance in Stühler et al (7), based on MS diagnosis. ^c^As in Stühler et al (7), alemtuzumab, cyclophosphamide, fingolimod,mitoxantrone, natalizumab, ocrelizumab,and rituximab.* | | | |

# **Table S13** Overview of outcomes in the sensitivity analysis of splitting the dataset in 2019

| **Outcome** | **Training set (N= 8159)**  median (interquartile range; range) or count (%) | **Test set (N= 1236)**  median (interquartile range; range) or count (%) |
| --- | --- | --- |
| CDP-free | 7235 (89) | 1179 (95) |
| Relapse-free | 6066 (74) | 1122 (91) |
| Number of relapses | 0 (1; 0-8) | 0 (0; 0-4) |
| *CDP confirmed disease progression; Number of relapses summarized by median (Interquartile range; range), the rest by count (%).* | | |

# **Table S14** Performance measures in the sensitivity analysis of splitting the dataset in 2019.

| **Outcome** | **Performance measure (95% CI)** | **10-fold cross-validation^a^ (N= 8159)** | **Temporal validation**  **(N= 1236)** |
| --- | --- | --- | --- |
| CDP | Calibration intercept | -0.056 (-0.128 to 0.016) | -0.099 (-0.369 to 0.171) |
|  | Calibration slope | 0.908 (0.827 to 0.989) | 1.778 (1.304 to 2.251) |
|  | C-index | 0.748 (0.734 to 0.762) | 0.833 (0.788 to 0.87) |
|  | MSE^b^ | 0.094 | 0.041 |
|  | RMSE% | 95% | 93% |
|  | Predicted proportion with CDP (range) | 0.12 (0 to 0.84) | 0.05 (0 to 0.24) |
|  | Observed proportion with CDP (range) | 0.11 (0 or 1) | 0.05 (0 or 1) |
| Relapse | Calibration intercept | 0.027 (-0.028 to 0.083) | -0.253 (-0.452 to -0.055) |
|  | Calibration slope | 0.553 (0.506 to 0.6) | 0.755 (0.513 to 0.997) |
|  | C-index | 0.675 (0.662 to 0.688) | 0.684 (0.64 to 0.726) |
|  | MSE | 1.328 | 0.143 |
|  | RMSE% | 149% | 100% |
|  | Mean predicted number of relapses (range) | 0.54 (0 to 32) | 0.14 (0 to 0.9) |
|  | Mean observed number of relapses (range) | 0.37 (0 to 8) | 0.11 (0 to 4) |
| *CDP confirmed disease progression; CI confidence interval; MSE mean squared error. ^a^The reported measures are estimated from out-of-sample predictions. ^b^Equivalent to the Brier score for binary outcomes; RMSE% relative percentage of root mean squared error.* | | | |


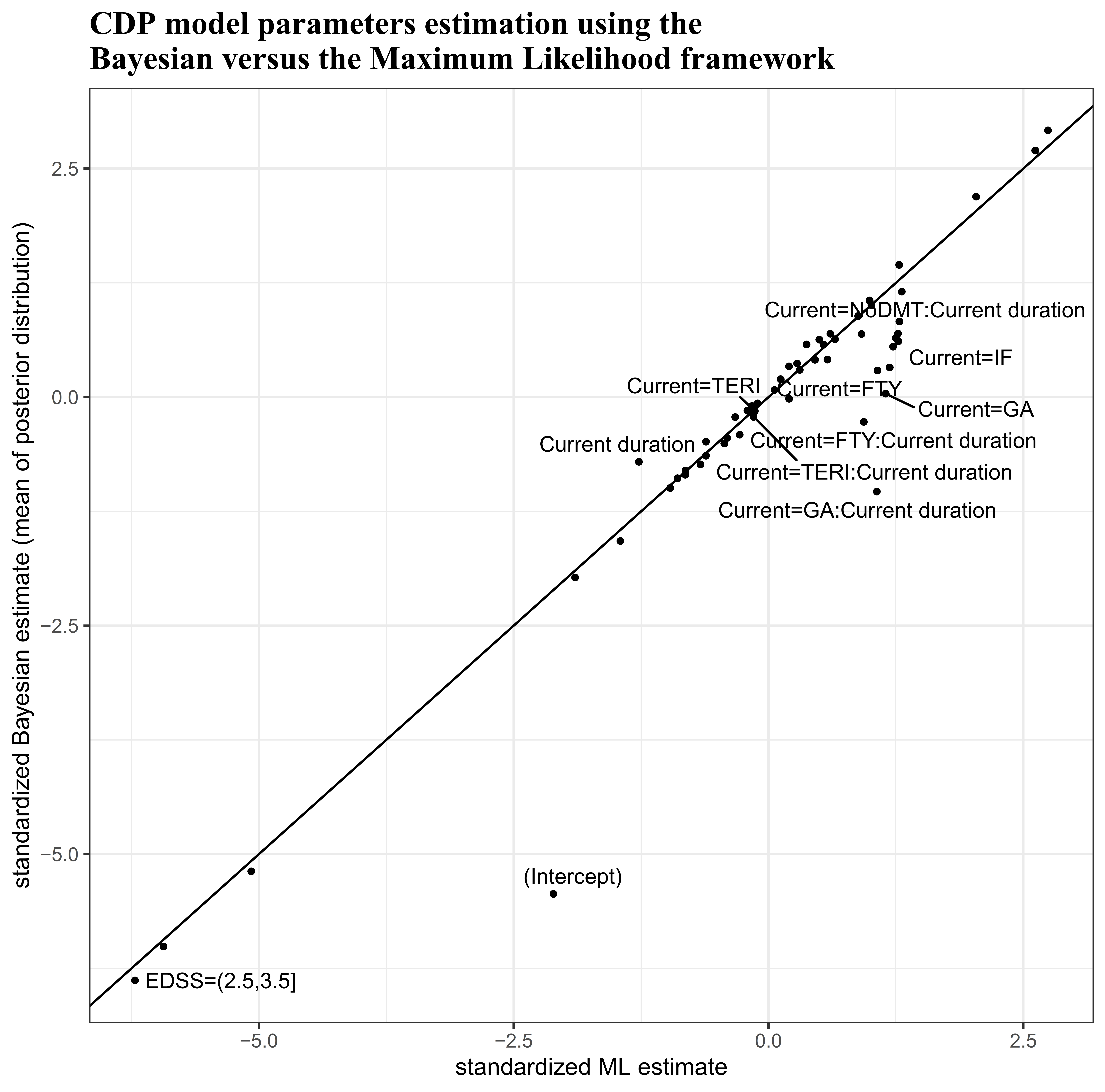


# **Fig.S9** Confirmed disease progression (CDP) model standardized parameters estimated in the main versus the sensitivity analysis.

*The coefficients for the current therapy and its interaction with current duration diverges from the diagonal indicating difference between the hierarchical Bayesian and maximum likelihood (ML) methods.*


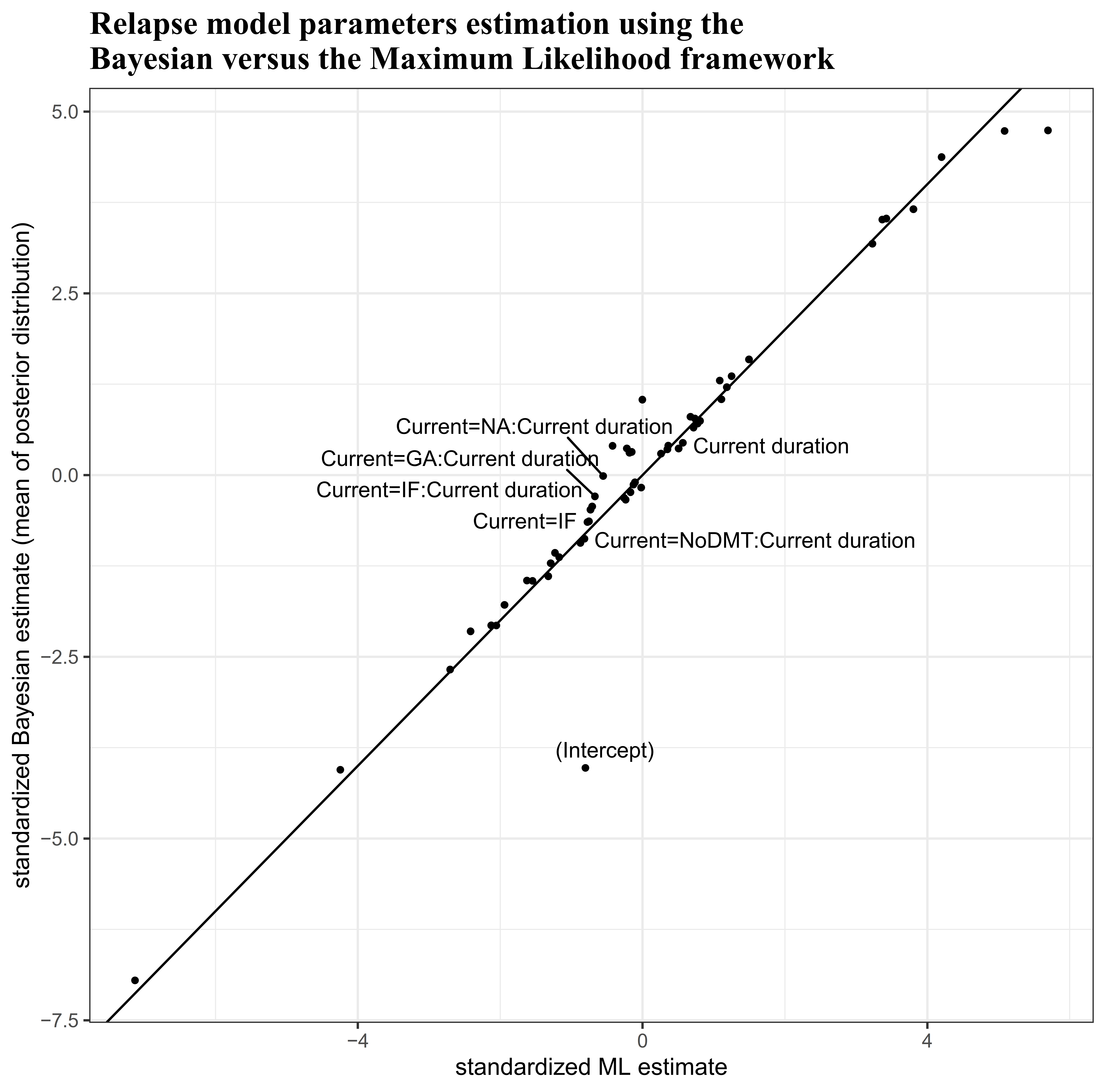


# **Fig.S10** Relapse model standardized parameters estimated in the main versus the sensitivity analysis.

*The coefficients for the current therapy and its interaction with current duration diverges from the diagonal indicating difference between the hierarchical Bayesian and maximum likelihood (ML) methods.*

# **Table S15** Performance measures in the sensitivity analysis with the fixed effects generalized linear models

| **Outcome** | **Performance measure (95% CI)** | **Temporal validation (95% CI)**  **(N= 3768)** |
| --- | --- | --- |
| CDP | Calibration intercept | 0.025 (-0.096 to 0.145) |
|  | Calibration slope | 0.697 (0.581 to 0.813) |
|  | C-index | 0.745 (0.717 to 0.772) |
|  | MSE^a^ | 0.073 |
|  | RMSE% | 99% |
|  | Predicted proportion with CDP (range) | 0.08 (0 to 0.6) |
|  | Observed proportion with CDP (range) | 0.08 (or 1) |
| Relapse | Calibration intercept | -0.418 (-0.512 to -0.325) |
|  | Calibration slope | 0.479 (0.391 to 0.567) |
|  | C-index | 0.637 (0.614 to 0.659) |
|  | MSE | 0.337 |
|  | RMSE% | 114% |
|  | Mean predicted number of relapses (range) | 0.33 (0 to 4.8) |
|  | Mean observed number of relapses (range) | 0.2 (0 to 4) |
| *CI confidence interval; CDP confirmed disease progression; MSE mean squared error. ^a^Equivalent to the Brier score for binary outcomes; RMSE% relative percentage of root mean squared error.* | | |
